# The dimer-monomer equilibrium of SARS-CoV-2 main protease is affected by small molecule inhibitors

**DOI:** 10.1038/s41598-021-88630-9

**Published:** 2021-04-29

**Authors:** Lucia Silvestrini, Norhan Belhaj, Lucia Comez, Yuri Gerelli, Antonino Lauria, Valeria Libera, Paolo Mariani, Paola Marzullo, Maria Grazia Ortore, Antonio Palumbo Piccionello, Caterina Petrillo, Lucrezia Savini, Alessandro Paciaroni, Francesco Spinozzi

**Affiliations:** 1 Department of Life and Environmental Sciences, New York Marche Structural Biology Center (NY-MaSBiC), Marche Polytechnic University, 60131 Ancona, Italy; 2 Department of Life and Environmental Sciences, Marche Polytechnic University, 60131 Ancona, Italy; 3 Department of Physics and Geology, CNR-IOM c/o University of Perugia, 06123 Perugia, Italy; 4 STEBICEF Department, University of Palermo, 90128 Palermo, Italy; 5 Department of Physics and Geology, University of Perugia, 06123 Perugia, Italy

**Keywords:** Biophysics, Computational biology and bioinformatics, Drug discovery, Molecular biology

## Abstract

The maturation of coronavirus SARS-CoV-2, which is the etiological agent at the origin of the COVID-19 pandemic, requires a main protease M^pro^ to cleave the virus-encoded polyproteins. Despite a wealth of experimental information already available, there is wide disagreement about the M^pro^ monomer-dimer equilibrium dissociation constant. Since the functional unit of M^pro^ is a homodimer, the detailed knowledge of the thermodynamics of this equilibrium is a key piece of information for possible therapeutic intervention, with small molecules interfering with dimerization being potential broad-spectrum antiviral drug leads. In the present study, we exploit Small Angle X-ray Scattering (SAXS) to investigate the structural features of SARS-CoV-2 M^pro^ in solution as a function of protein concentration and temperature. A detailed thermodynamic picture of the monomer-dimer equilibrium is derived, together with the temperature-dependent value of the dissociation constant. SAXS is also used to study how the M^pro^ dissociation process is affected by small inhibitors selected by virtual screening. We find that these inhibitors affect dimerization and enzymatic activity to a different extent and sometimes in an opposite way, likely due to the different molecular mechanisms underlying the two processes. The M^pro^ residues that emerge as key to optimize both dissociation and enzymatic activity inhibition are discussed.

## Introduction

The COVID-19 pandemic is the ongoing worldwide health emergency caused by the coronavirus SARS-CoV-2 (severe acute respiratory syndrome-cororavirus-2)^1,2^. Coronaviruses (CoVs) are enveloped positive-stranded RNA viruses; once the virion gets into the cell, the single-strand RNA translates into two overlapping polyproteins, termed pp1a and pp1ab, which mediate viral replication and proliferation. The virus maturation involves a highly complex cascade of proteolytic processing events on these polyproteins: most cleavage events are ruled by a nonstructural protein, the CoV main protease (M^pro^, also known as ${{3{\text{CL}}^{\mathrm{pro}}}}$), a three-domain (domains I to III) protein^3^. The enzyme shows first autolytic cleavage from pp1a and pp1ab, then starts processing the two polyproteins at no less than 11 conserved sites^3^.

Because of this mechanism of action, inhibiting M^pro^ might lead to an attenuation of the viral infection. Indeed, this enzyme is a very attractive target for anti-CoV drug design: the M^pro^ sequence is highly conserved among various CoVs^4^, as mutations of M^pro^ turn out to be often fatal for the virus^5^. Thus, the risk of mutation-mediated drug resistance is very low and inhibitors will display broad-spectrum antiviral activity. In addition, M^pro^ inhibitors are unlikely to be toxic because human proteases have different cleavage specificity. A second point should be however considered: the published X-ray structures of SARS-CoV-2 M^pro^, obtained both in the presence and in the absence of inhibitors^6,7^, revealed that two M^pro^ monomers form a functional active homodimer, as already detected in different coronaviruses^3^, which share with SARS-CoV M^pro^ almost all the amino-acids involved in the dimerization. In such homodimer, the two monomers are arranged almost perpendicular to each other^7^ and each monomer comprises the catalytic dyad His41-Cys145 and the substrate-binding site located in a cleft between domains I and II. Domain III, which contains five $\alpha$-helices arranged into a globular cluster, is directly involved in controlling the dimerization of M^pro^ mainly through a salt-bridge between Glu290 of one monomer and Arg4 of the other^8^. Quite remarkably, while individual monomers are enzymatically inactive, M^pro^ is active in the dimeric form. The structural reason behind the functionality of the dimer is probably due to the interaction of the N-finger of each of the two monomers with Glu166 of the other monomer, which establishes the shape of the so-called S1 pocket of the substrate-binding site^9^. To approach this interaction site, the N-terminal amino acid residues are squeezed in between domains II and III of the parent monomer and domain II of the other monomer^7^.

According to these considerations, two different strategies have been considered for the development of therapeutic agents: first, direct inhibition of the catalytic site by using molecules targeting the substrate binding pocket; second, attenuation of the catalytic activity by using inhibitors targeting the dimerization site. The second alternative is strictly related to the M^pro^ equilibrium between dimers and monomers in solution. The thermodynamic equilibrium of M^pro^ dissociation process has been recently studied by analytical ultracentrifugation. Sedimentation velocity experiments provided a value of about 2.5 $\mu$M for the apparent dimer dissociation constant $K_D$^7^. However, a more recent estimate by mass-spectrometry based assays established for $K_D$ a much lower value of $0.14 \pm 0.03$ $\mu$M, indicating that M^pro^ has a stronger preference to dimerize in solution than expected. In the case of SARS-CoV M^pro^, an even wider discrepancy among the different estimates of the dimer-monomer dissociation constants has been observed, with the values of $K_D$ provided by various experimental techniques falling in a range from $230 \pm 30$ $\mu$M^10^ down to $0.19 \pm 0.03$ $\mu$M^11^. In this framework, synchrotron Small Angle X-ray Scattering (SAXS) can be a very sharp method to determine M^pro^ dimer-monomer equilibrium in solution. In fact, beyond pioneering SAXS studies performed also by some of us to investigate the thermodynamic features related to $\beta$-lactoglobulin dimerization^12–15^, this approach has more recently provided noticeable information in many issues^16,17^. Among them it is worth citing the case of A3G, a key enzyme for HIV-1 infection^18^, of LRRK2 protein, linked to Parkinson’s disease^19^. Hence, given the above mentioned uncertainty on the $K_D$ value that rules M^pro^ dimerization, we decided to take advantage of SAXS to provide new insights on the SARS-CoV-2 M^pro^ dimer-monomer equilibrium. The study was performed both in the absence and in the presence of a set of in-silico selected small inhibitors, whose activity was spectroscopically assayed, in order to simultaneously test their therapeutic potential with respect to dimerization inhibition. By measuring the large-scale structural features of SARS-CoV-2 M^pro^ as a function of temperature, protein concentration and in the presence of different amounts of inhibitors we provide an accurate thermodynamic picture of the SARS-CoV-2 M^pro^ inhibitor-dependent dimerization process.

## Results

**Figure 1:**
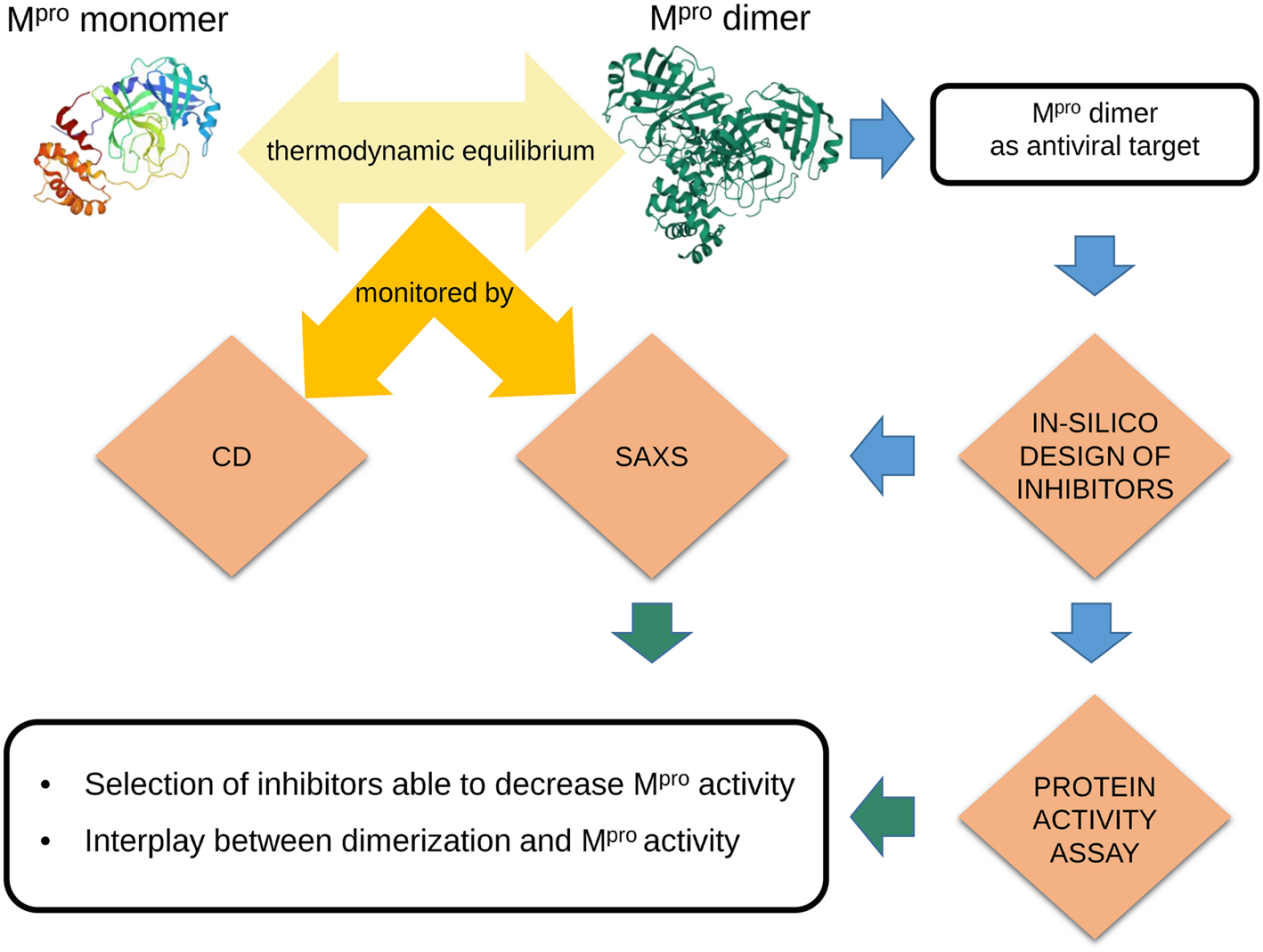
Graphic display of the flowchart of the biophysical method described in the text.

Our biophysical multi-technique approach, mainly based on SAXS, by which we studied the effects of potential inhibitors of the SARS-CoV-2 M^pro^ on the dimerization process and the connection with the catalytic activity, is reported in the flowchart shown in Fig. 1. We have first derived the thermodynamic parameters controlling the M^pro^ dimer-monomer equilibrium in solution by SAXS and CD spectroscopy techniques in the absence of inhibitors. Considering the results obtained by Graziano et al.^20^ for the SARS-CoV M^pro^, we have chosen to investigate a range of protein concentrations from 3 and 30 $\mu$M, the molarity being expressed in terms of M^pro^ monomers. It should be noted that, using these protein concentrations, one can discriminate between values of the dissociation constant that fall in the quite wide range of $\approx 0.2-200$ $\mu$M (see Eq. 2).

Subsequently, we have studied by SAXS experiments the M^pro^ dimer-monomer equilibrium in the presence of a series of potential inhibitors, selected from an *in-house* database containing commercial and synthetic compounds. Just one protein concentration, 30 $\mu$M, and two concentrations of inhibitors, 30 and 60 $\mu$M, corresponding to an inhibitors-to-monomer M^pro^ molar ratio of 1 and 2, have been investigated. Activity assays were also performed and results are correlated with the M^pro^ dimerization inhibition.

### M^pro^ dimerization and thermal stability

The dimer-monomer equilibrium of SARS-CoV-2 M^pro^ has been investigated at different protein concentrations by performing in-solution SAXS experiments in the temperature range between 15$^\circ$ and 45$^\circ$ C and far-UV CD measurements at room temperature. Far-UV CD spectroscopy was also used to study the M^pro^ thermal stability, monitoring the unfolding transition between 10$^\circ$ and 80$^\circ$ C.

#### SAXS

**Figure 2:**
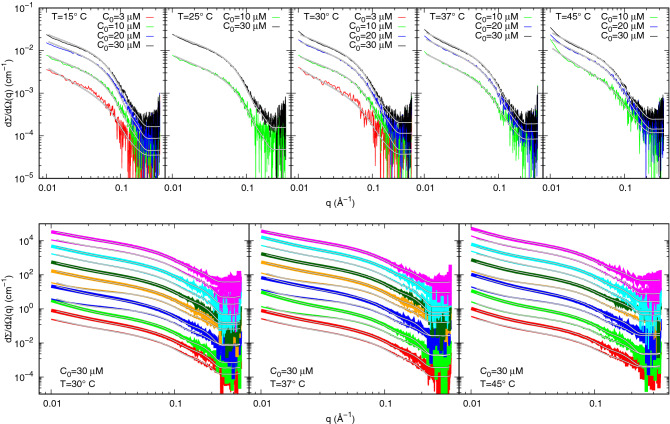
SAXS data and fits. **Top panels**: SAXS experimental data of SARS-CoV-2 M^pro^ without inhibitors and best theoretical fits obtained by GENFIT software^21,22^ (solid black and white lines). Each panel reports a dataset obtained at the same temperature, as shown in the top left corner, and at different nominal protein concentration $C_\circ$. **Bottom panels**: SAXS data of M^pro^ at fixed concentration $C_\circ$=30 $\mu$M in the presence of inhibitors. Each panel reports the curves at the same temperature, shown in the bottom left corner. Red, green, blue, orange, dark-green, cyan and magenta refers to inhibitor **1**, **2**, **3**, **4**, **5**, **6** and **7**, respectively. Thin and thick lines refer to inhibitor concentrations $C_{\mathrm{I}}$ of 30 and 60 $\mu$M, respectively. Subsequent curves are multiplied by a factor 3.0 for clarity. Solid black and white lines are the best fits obtained by GENFIT^21^.

SAXS data of SARS-CoV-2 M^pro^ recorded at the B21 beam-line of the Diamond Synchrotron (Didctot, UK) at different protein concentrations and temperatures are shown as log-log plots in Fig. 2, top panels.

We have assumed that SAXS curves arise from both M^pro^ monomer and dimer species, according to the thermodynamic equilibrium dissociation process given by the relationship:

1 $$\begin{aligned} {{\text{M}}^{{\text{pro}}}_{2}} \rightleftharpoons 2 {{\text{M}}^{{\text{pro}}}_{1}}, \end{aligned}$$

The corresponding equilibrium dissociation constant is

2 $$\begin{aligned} K_{D}=\frac{[{{\text{M}}^{{\rm pro}}_{1}}]^2}{[{{\text{M}}^{{\rm pro}}_{2}}]} = \frac{2 C x_1^2}{1-x_1} = e^{-\Delta G_{D}/({\text{R}}T)}, \end{aligned}$$

where *C* is the total molar concentration of monomers, $x_1$ is the molar fraction of proteins that remain in the monomeric state, $\Delta G_{D}$ is the dissociation Gibbs free energy change, ${\mathrm{R}}$ is the universal gas constant and *T* the absolute temperature. To note, Eq. (2) can be solved in terms of $x_1$,

3 $$\begin{aligned} x_1=K_D\frac{\sqrt{1+8 C/K_D}-1}{4 C} . \end{aligned}$$

According to classical thermodynamics, the temperature dependence of $\Delta G_{D}$ is

4 $$\begin{aligned} \Delta G_{D}=\Delta G_{D}^\circ +(\Delta {C_p}_{D}-\Delta S^\circ _D)(T-T_\circ )-\Delta {C_p}_{D} T \log \frac{T}{T_\circ } \end{aligned}$$

where $\Delta G_{D}^\circ =-\mathrm{R}T_\circ \log K^\circ _D$ is the dissociation Gibbs free energy at the reference temperature $T_\circ =298.15$ K ($K^\circ _D$ being the associated equilibrium constant), $\Delta {C_p}_{D}$ is the change of the constant pressure heat capacity upon dissociation (here supposed independent on *T*) and $\Delta S_{D}^\circ$ is the dissociation entropy at $T_\circ$.

The macroscopic differential scattering cross section, which is the experimental information provided by a SAXS curve, for a system of interacting monomers and dimers can be written as

5 $$\begin{aligned} \frac{d\Sigma }{d\Omega }(q)=N_A\kappa C_N P(q)S_M(q)+B, \end{aligned}$$

$N_A$ being Avogadro’s number, $\kappa$ an unknown fraction of the nominal protein molar concentration $C_N$ ($C=\kappa C_N$), *B* an arbitrary flat background that takes into account possible uncertainties in the determination of transmissions of proteins and buffers samples. *P*(*q*) represents the average form factor of the system

6 $$\begin{aligned} P(q)=x_1P_1(q)+\frac{1}{2}(1-x_1)P_2(q), \end{aligned}$$

where $P_j(q)$ stands for the form factor (which is the orientational average of the excess squared X-ray scattering amplitude) of the M^pro^ monomer ($j=1$) or dimer ($j=2$). We have calculated $P_j(q)$ from the the crystal structure of SARS-CoV-2 M^pro^ dimer recently determined^7^ (PDB code 6y2e) considering one chain ($j=1$) or both chains ($j=2$) by means of the SASMOL method^23^. This method takes into account the contribution to the scattering due to the hydration water molecules around the protein, whose positions are found by embedding the atomic structure in a tetrahedral close packed lattice. For SARS-CoV-2 M^pro^ monomer and dimer, 726 and 1243 hydration water molecules have been respectively calculated, suggesting that for the dimer formation about 200 water molecules are removed from the hydration shell of both monomers. Hence, the water molecules attributed to each monomer decrease from 726 to 621 upon M^pro^ dimerization. This indicates that the dimerization process is accompanied with slight structural changes reducing the average area accessible to solvent. The $S_M(q)$ term in Eq. (5) is the so-called “measured” structure factor, which describes the long range intermolecular interactions among all the particles in solution. For sake of simplicity, here we consider a common effective structure factor that takes into account monomer-monomer, monomer-dimer and dimer-dimer interactions. Considering that at low *q* all the experimental scattering curves (Fig. 2 top panels) show a positive deviation from a Guinier trend, indicative of the prevalence of protein-protein attraction with respect to repulsion, we have approximated the structure factor by the one of fractal distribution of inhomogeneities developed by Teixeira^24^, whose main parameters are *D*, the fractal dimension of the aggregates, $r_0$, the effective radius of the aggregating protein molecule and $\xi$, the correlation length, which can be interpreted as the average size of the aggregates (see Eqs. 10, 11 and 12).

The above described model, which combines SARS-CoV-2 M^pro^ thermodynamic and structural features, has been adopted to simultaneously analyze the whole set of the SAXS curves, recorded at different temperatures and concentrations, shown in Fig. 2, top panels. Fitting parameters shared by all the curves are $K^\circ _D$, the dissociation equilibrium constant at $T_\circ$, $\Delta {C_p}_{D}$, the constant pressure heat capacity upon dissociation, $\Delta S_{D}^\circ$ and the dissociation entropy at $T_\circ$. Another parameter common to all the curves is the relative mass density of the hydration water (in general higher than 1), $d_h$, which is taken into account in the SASMOL method^23^. The shared fitted parameters are shown in Table 1, while all the other are reported in Supplementary Table S1.

**Table 1:** Thermodynamic parameters resulting from the global fit of SAXS data for SARS-CoV-2 M^pro^ without inhibitors at different temperatures and concentrations.

$K_D^\circ$	($\mu$M)	7 ± 1
$\Delta {C_p}_D$	(kJ $\hbox {mol}^{-1}$ $\hbox {K}^{-1}$)	1.7 ± 0.7
$\Delta S^\circ _D$	(J $\hbox {mol}^{-1}$ $\hbox {K}^{-1}$)	50 ± 20
$d_h$		1.100 ± 0.006

The most important parameter obtained by the simultaneous fit of SAXS data is the dissociation constant $K^\circ _D$, which turns out to be $7 \pm 1$ $\mu$M, in good agreement with the value obtained by Graziano et al.^20^ on the very similar main protease from SARS-CoV. The corresponding dissociation Gibbs free energy (calculated with Eq. 2) is $\Delta G_{D}^\circ \simeq 30$ kJ $\hbox {mol}^{-1}$, a value quite similar to the one observed for the $\beta$-lactoglobulin dimer dissociation at neutral pH^25^. Regarding the dissociation entropy, we have obtained a positive value, $50 \pm 20$ J $\hbox {mol}^{-1}$ $\hbox {K}^{-1}$, meaningfully smaller with respect to the one derived for the above mentioned $\beta$-lactoglobulin case^25^. It should be noticed that in a dissociation process, many factors besides translational and rotational motions contribute to a positive dissociation entropy and it is difficult to separate them. One such factor is certainly the removal of about 200 hydration water molecules from the monomer-monomer interface when the dimer is formed. The change of the heat capacity at constant pressure upon dissociation resulted positive and large. This parameter indirectly describes the monomer-monomer interface, as it can be attributed to the hydration and correlates with the interface size^26^. The set of parameters reported in Table 1 allows to calculate the M^pro^ equilibrium dissociation constant, together with its standard deviation, at any temperature. Results are shown in Supplementary Fig. S1, top left panel. We notice a slight increase of $K_D$ with *T*, an effect that is mainly due to the large increase of the constant pressure heat capacity upon dissociation. However, a further investigation on the monomer-monomer interface area and its relationship with the dissociation heat capacity^27^ requires further calorimetric experiments in order to obtain lower estimation errors. Finally, the relative density of the hydration shell is little more than one, in agreement with previous literature results on globular proteins^28–30^. The determination of the thermodynamic features of the dimer-monomer equilibrium of M^pro^, in conditions quite similar to those found *in vivo*, is a fundamental step to investigate the effects of drugs aimed to inhibit dimerization and underlines the importance to further investigate M^pro^ monomer-monomer interface by in-solution techniques.

#### Far-UV CD

**Figure 3:**
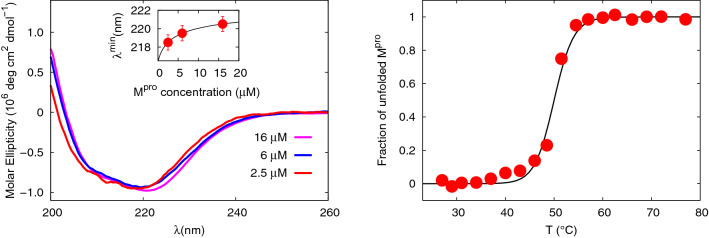
CD data and fits. **Left**: far-UV CD spectra of SARS-CoV-2 M^pro^ at three different concentrations. The CD data are represented in molar ellipticity units. Inset: position of the minimum of the spectra as a function of the concentration (red circles). The continuous line represents an estimate of the minimum position based on Eq. (7). Results from the fit are: $\lambda ^{\mathrm{min}}_{\mathrm{mon}}=216.4\pm 0.1$ nm and $\lambda ^{\mathrm{min}}_{\mathrm{dim}}=222.9\pm 0.1$ nm. **Right**: Thermal melting of the SARS-CoV-2 M^pro^ (16 $\mu$M concentration) followed by monitoring the far-UV CD signal at 221 nm. The continuous line results from the theoretical fitting model arising from Eq. (8).

To provide further insights on the dimer-monomer equilibrium, we have measured the far-UV CD spectra of M^pro^ at three different concentrations, as shown in Fig. 3, left panel.

At the higher concentration of  16 $\mu$M, the ellipticity shows a minimum wavelength $\lambda ^{\mathrm{min}}$ at about 221 nm and a shoulder centered at about 208 nm, which are typical of proteins with $\alpha$-helical and $\beta$-sheet content^31,32^, fully consistent with the structural features of the SARS-CoV-2 M^pro^^7^, in agreement with CD measurements of the same enzyme^33^ and of the very much similar SARS-CoV M^pro^^34^. As concentration decreases, $\lambda ^{\mathrm{min}}$ shifts towards lower values, thus reporting an increase of the $\beta$-sheet component at the expense of the $\alpha$-helical content^31^. Quite interestingly, if we take the dissociation constant of $K_D=7 \pm 1$ $\mu$M, in the concentration range from 2.5 to 16 $\mu$M a decrease of about a factor 2 in the fraction of monomers is expected (from around 0.67 to 0.36, see Eq. 3). This large shift in the dimer-monomer populations can reasonably yield the changes occurring to the M^pro^ secondary structure and revealed by CD spectroscopy. Within this working hypothesis, we can describe the $\lambda ^{\mathrm{min}}$ trend in terms of the dimer-monomer equilibrium through the following expression:

7 $$\begin{aligned} \lambda ^{\mathrm{min}}=x_1\lambda ^{\mathrm{min}}_{\mathrm{mon}}+(1-x_1)\lambda ^{\mathrm{min}}_{\mathrm{dim}} \end{aligned}$$

where we take a fixed value of $K_D=7$ $\mu$M as estimated by SAXS, while $\lambda ^{\mathrm{min}}_{\mathrm{mon}}$ and $\lambda ^{\mathrm{min}}_{\mathrm{dim}}$ are the minimum wavelength parameters corresponding to the monomer and the dimer spectra, respectively. As shown in the inset of Fig. 3 (left panel), the trend of the $\lambda ^{\mathrm{min}}$ values is fitted in an excellent way with Eq. (7).

The thermal stability of the M^pro^ has been characterized by monitoring the signal at 221 nm of the M^pro^ sample at 16 $\mu$M concentration, within the simplified hypothesis that the melting curve arises mainly from dimers. The rather sharp transition we have obtained is shown in Fig. 3 (right panel) and clearly suggests a two-state model, where the dimer unfolds and yields two random-coil monomeric chains:

8 $$\begin{aligned} {{\text{M}}^{{\text{pro}}}_{2}} \rightleftharpoons 2 {{{\text{M}}^{{\text{pro}}}_{1,{{\text{unf}}}}}}.  \end{aligned}$$

Considering the scheme 8, if we hypothesize that the dimer can unfold to two random-coil monomeric chains, we obtain an apparent melting temperature $T_m$ of $50^\circ$ C, with a melting Van’t Hoff enthalpy of $\Delta H_{\mathrm{v}}= 810 \pm 60$  kJ/mol. This value is in good agreement with the Van’t Hoff enthalpy $\Delta H_{\mathrm{v}} \sim 880$ kJ/mol estimated through the equation $\Delta H_{\mathrm{v}}=4\mathrm{R}T_m^2{C_{p,\mathrm max}}/ \Delta H_{\mathrm{cal}}$ from DSC measurements^33^. It is also worth of note that, by taking $\Delta H_{\mathrm{cal}}=443$ kJ/mol^33^, it turns out a ratio $\Delta H_{\mathrm{v}}/\Delta H_{\mathrm{cal}}\sim 1.8$: such a value larger than 1 is fully consistent with the unfolding transition coupled to the dimer dissociation. Quite interestingly, the thermal stability as revealed by CD measurements supports a view where about 90 the folded state in the temperature range investigated by SAXS experiments, i.e. up to $45^\circ$ C, thus validating the model we used to interpret the corresponding scattering curves.

### M^pro^ dimer-monomer equilibrium in presence of inhibitors

#### *In-silico* inhibitor selection

To identify new inhibitors of SARS-CoV-2 main protease from a large *in-house* database, we applied the *in silico* protocol, recently proposed by some of us^35^. The flowchart of the adopted protocol is depicted in Supplementary Fig. S2. As a first step, we performed molecular docking studies on the compounds present in the database to analyze their binding capability in the catalytic active site of the SARS-CoV-2 M^pro^ (PDB code 6y2f)^7^, as detailed in the Materials and Methods section. Supplementary Fig. S3 shows the 3D binding active site of SARS-CoV-2 M^pro^ co-crystallized with the native inhibitor **13b**^7^ covalently bonded to Cys145. The ligand binds to the enzymatic catalytic cleft of the protease located between domains I and II. The 3D binding site representation (Supplementary Fig. S3) highlights the interactions with the amino acid residues involved in the inhibition mechanism, such as Met49, Met165, Glu166, His164, Phe140, Gly143 and the catalytic Cys145. It is noteworthy the presence of hydrogen bonds between the pyridone moiety of ligand and Glu166, which rules the catalytic activity driving the SARS-CoV-2 M^pro^ to adopt an inactive conformation. The resulting best docked molecules have been selected based on a docking score cut-off of $-6.5$ kcal/mol and submitted to ligand based approaches, by taking advantage of the web-service DRUDIT (DRUgs Discovery Tools), an open access virtual screening platform recently developed^36^, which represents the evolution of previous well-established protocols based on molecular descriptors^37,38^.

DRUDIT implements the ligand based template of SARS-CoV-2 M^pro^, available in the Biotarget Finder tool, which has been recently proposed as a useful mean in the identification of new SARS-CoV-2 M^pro^ modulators. Subsequently, the ligands selected by molecular docking were submitted to DRUDIT, as elsewhere reported^35^, allowing the evaluation of their affinity to SARS-CoV-2 M^pro^ by the values of Drudit Affinity Score (DAS). The features of the ligand-based approaches based on molecular descriptors enabled us to assess topological, thermodynamic and charge-related characteristics of the ligands. Thus, two complementary standpoints in the evaluation of the binding capability (ligand- and structure-based) covered all the interaction aspects in the ligand-target complex. The top scored molecules (selected based on a DAS cut-off of 0.65) were processed by Induced Fit Docking (IFD) calculations to further screen the hits to submit to *in-wet* test. In Supplementary Fig. S4 and in Table 2 the seven best scored structures are reported. The analysis of the results in Table 2 shows as the selected compounds present similar overall scores (${\mathrm{IFD\_score}}$). This confirms the robustness of the ligand-based approach exploited by DRUDIT, which is able to give an account of the receptor-ligand binding although it is based on molecular descriptors that, as known, do not take into consideration the 3D shape of the binding site.

**Table 2:** IFD results for the seven selected inhibitors compared with the **13b** compound.

Inhibitor	${\mathrm{Prime\_Energy}}$	${\mathrm{XPG\_score}}$	${\mathrm{IFD\_score}}$
**1**	$-11527.6$	$-8.580$	$-584.958$
**2**	$-11360.0$	$-10.772$	$-578.774$
**3**	$-11674.5$	$-7.895$	$-591.618$
**4**	$-11497.2$	$-5.969$	$-580.827$
**5**	$-11517.1$	$-8.918$	$-584.772$
**6**	$-11489.6$	$-9.176$	$-583.655$
**7**	$-11561.2$	$-10.409$	$-588.468$
**13b**	$-11736.3$	$-7.944$	$-594.758$

**Figure 4:**
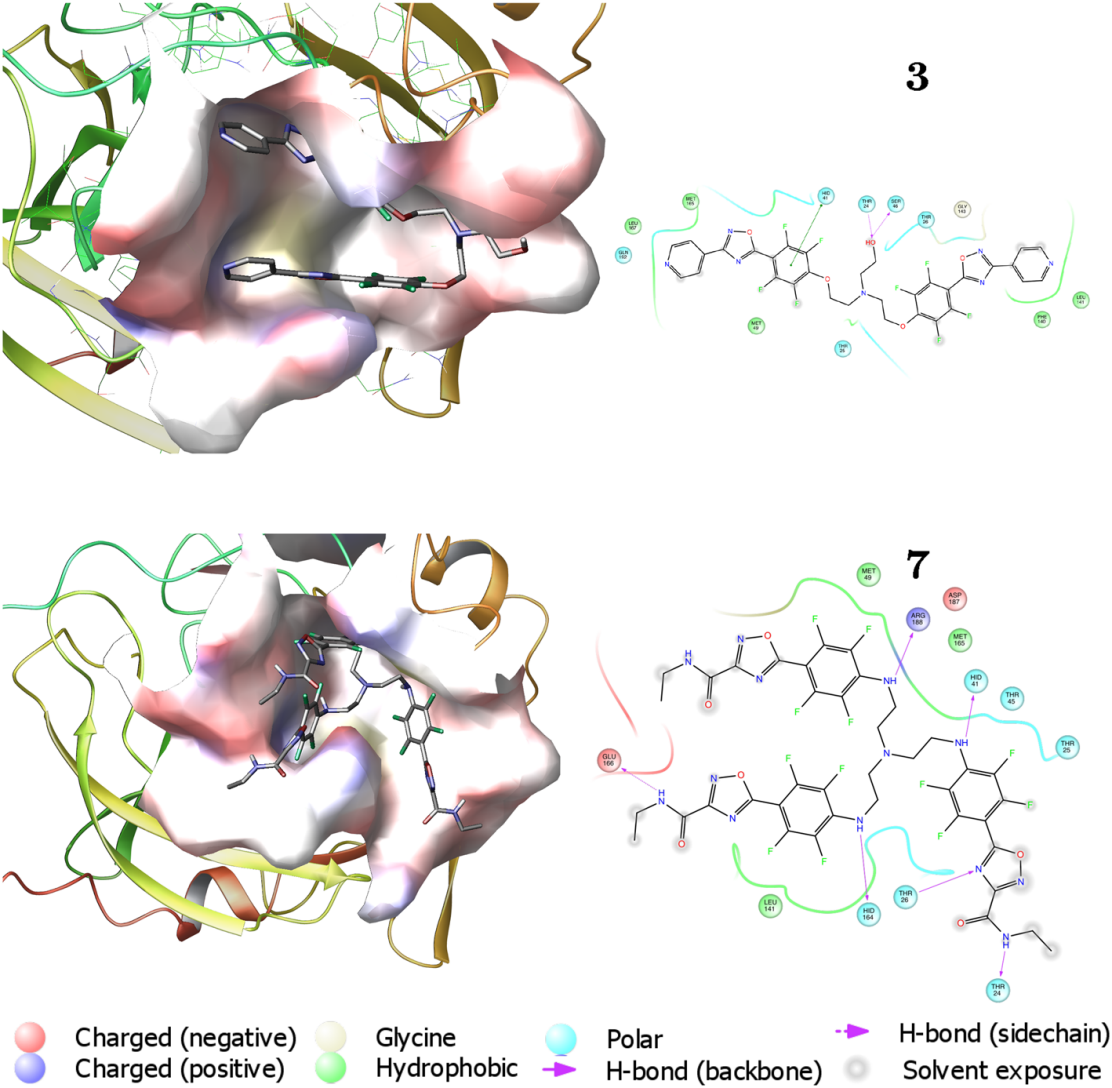
3D binding modes of best scored compounds **3** and **7** into SARS-CoV-2 M^pro^ active site (**left**) and corresponding amino acid maps (**right**). The picture is elaborated by Maestro Schrödinger, version 10.2 (2017)^39^.

Figure 4 reports the first two best scored molecules **3** and **7** (according to the ${\mathrm{IFD\_score}}$ parameter) in the binding site (left panel) and their related amino-acid maps (right panel). The two molecules are deeply buried in the cleft of the substrate-binding pocket, but unlike the co-crystallized ligand **13b**, they interact with a somehow different pattern of amino-acids. This evidence suggests that these compounds are not covalently bound to the SARS-CoV-2 M^pro^ catalytic site.

#### M^pro^ activity assays

**Figure 5:**
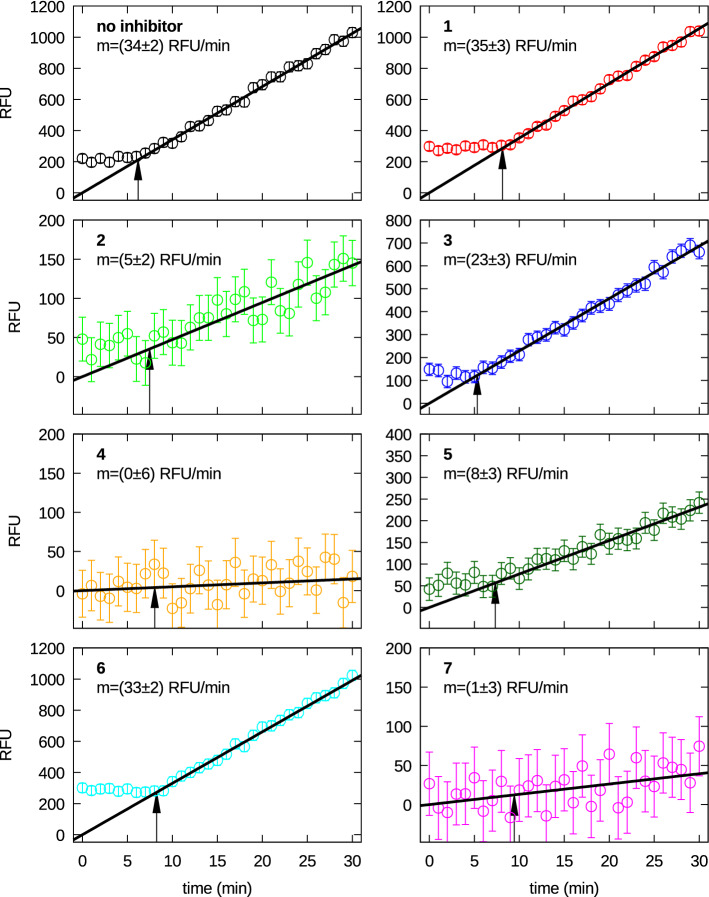
Fluorescence inhibition curves of the selected compounds, as indicated in each frame. The straight lines are the best fitting lines obtained considering data points comprised between the time indicated by the arrow and 30 min. The slope of the straight line is reported in each frame.

**Figure 6:**
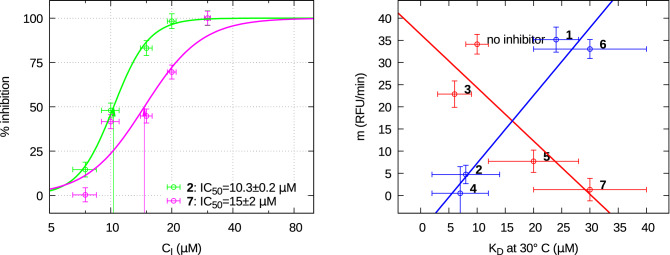
**Left**: percent inhibition data of SARS-CoV-2 M^pro^ as a function of the concentration of inhibitor **2** (green points) and **7** (magenta points). Best fits with the Hill equation are shown as solid lines. **Right**: correlation map between the catalytic activity, represented by the RFU slope *m*, and dimer dissociation capability, measured by the dissociation constant $K_D$ at $30^\circ$ C, of the seven SARS-CoV-2 M^pro^ inhibitors at $C_{\mathrm{I}}=60$ $\mu$M.

The selected inhibitors have been tested for their efficacy to reduce the M^pro^ activity. As reported in Fig. 5, the time dependence of substrate fluorescence after hydrolysis indicates that the catalytic activity of M^pro^ changes in the presence of the selected compounds. In particular, compounds **2**, **4**, **5** and **7** induced an irreversible inactivation of the enzyme, while compounds **1** and **6** resulted rather inactive. For two of the most effective compounds (**2** and **7**) inhibition tests have been carried out as a function of the concentration. Unfortunately, we have not been able to perform this test for compound **4**, which shows the best inhibition efficacy, as it produces a fluorescence signal that partially obscures that of the substrate. Results are shown in Fig. 6 (left panel). Percent inhibition data have been fitted with the Hill equation, $p(C_{\mathrm{I}})=100/(1+(\mathrm{IC}_{50}/C_{\mathrm{I}})^n)$, to get the half maximal effective concentration, ${\mathrm{IC}_{50}}$, and the Hill slope *n*. We obtained ${\mathrm{IC}}_{50} =10.3 \pm 0.2$ $\mu$M for **2** and $15 \pm 2$ $\mu$M for **7**, with $n=5 \pm 1$ and $3 \pm 1$, respectively. These values of *n* larger than one indicate that the binding is positively cooperative, in agreement with other recent experimental results^40^.

#### SAXS

SAXS curves of SARS-CoV-2 M^pro^ at nominal concentration $C_\circ =30$ $\mu$M and in the presence of each of the seven selected potential inhibitors, at concentrations $C_{\mathrm{I}}=30$ $\mu$M (thin lines) or 60 $\mu$M (thick lines), are reported in the bottom panels of Fig. 2 as log-log plots. Each panel refers to a different temperature, as indicated. Unfortunately, some of the foreseen conditions are missing (e.g. SAXS curves of inhibitor 5 at 60 $\mu$M), due to an experimental problem with the sample injection in the beam-line capillary. SAXS data have been analysed with the same approach adopted for data without inhibitors, with the further assumption that, for each compound, the thermodynamic parameters are linear functions of its concentration $C_{\mathrm{I}}$, namely $\Delta G^\circ _D = \Delta G^\circ _{D,0}(1+\alpha _G C_{\mathrm{I}})$, $\Delta {C_p}_D = \Delta {C_p}_{D,0}(1+\alpha _{C_p} C_{\mathrm{I}})$, and $\Delta S^\circ _D = \Delta S^\circ _{D,0}(1+\alpha _S C_{\mathrm{I}})$. The three terms $\Delta G^\circ _{D,0}=-\mathrm{R}T_\circ \log K^\circ _{D,0}$, $\Delta {C_p}_{D,0}$ and $\Delta S^\circ _{D,0}$ are exactly the values already obtained from the analysis of SAXS data without inhibitors (reported in Table 1), and the three corresponding constant rates $\alpha _G$, $\alpha _{C_p}$ and $\alpha _S$ are fitting parameters common to all the SAXS curves corresponding to the same inhibitor. The high quality of the fitting procedure can be appreciated in Fig. 2 (bottom panels), where the calculated SAXS curves are superposed to the experimental ones and the resulting thermodynamic common fitting parameters are shown in Table 3, first panel.

**Table 3:** **Top panel**: common thermodynamic fitting parameters of the analysis of SAXS data for SARS-CoV-2 M^pro^ samples with inhibitors. **Middle and bottom panels**: dissociation constants derived by the analysis of SAXS data for SARS-CoV-2 M^pro^ samples with inhibitors.

Inhibitor	$\alpha _G$	$\alpha _{C_p}$	$\alpha _S$					
	($10^{-2}$ $\mu \hbox {M}^{-1}$)	($10^{-2}$ $\mu \hbox {M}^{-1}$)	($10^{-2}$ $\mu \hbox {M}^{-1}$)					
**1**	$-0.18\pm 0.02$	$-6\pm 3$	$-4\pm 1$					
**2**	$-0.0\pm 0.1$	$-1\pm 3$	$-4\pm 1$					
**3**	$0.00\pm 0.08$	$0\pm 4$	$-8\pm 4$					
**4**	$0.0\pm 0.1$	$-2\pm 2$	$-1\pm 5$					
**5**	$-0.14\pm 0.05$	$-1\pm 4$	$-4\pm 4$					
**6**	$-0.20\pm 0.05$	$-6\pm 3$	$-1.7\pm 0.8$					
**7**	$-0.19\pm 0.07$	$-3\pm 3$	$-5\pm 2$					

30 $\mu$M Inhibitor		**1**	**2**	**3**	**4**	**6**	**7**	
$K_D^\circ$	($\mu$M)	$14\pm 1$	$8 \pm 3$	$7\pm 2$	$6 \pm 2$	$15\pm 3$	$14\pm 3$	

60 $\mu$M Inhibitor		**1**	**2**	**3**	**4**	**5**	**6**	**7**
$K_D^\circ$	($\mu$M)	$26\pm 4$	$8 \pm 6$	$7 \pm 4$	$5 \pm 4$	$19\pm 7$	$30\pm 10$	$30\pm 10$

The inhibitors with the lowest negative values of $\alpha _G$ (Table 3, first panel) are those that mostly favour dimer dissociation. Results reported in Table 3 suggest that compounds **1**, **6**, and **7** are, within the experimental error, mostly able to increase the dissociation equilibrium constant, which at $C_{\mathrm{I}}=30$ $\mu$M becomes as large as $\approx 15$ $\mu$M and, at $C_{\mathrm{I}}=60$ $\mu$M almost doubles its value, reaching $\approx 30$ $\mu$M. We indeed recall that, in the absence of inhibitors, the value of $K^\circ _{D,0}$ is $7 \pm 1$ $\mu$M (Table 1). Inhibitor **5** is slightly less active: at $C_{\mathrm{I}}=60$ $\mu$M we found a dissociation equilibrium constant of $\approx 20$ $\mu$M. The other three compounds, namely **2**, **3** and **4**, show a value of $\alpha _G$ close to 0, indicating that they do not affect in a significant way the dimer-monomer equilibrium of M^pro^. Despite the high uncertainties on $\alpha _{C_p}$ and $\alpha _S$, their negative values suggest that upon dissociation there are changes of heat capacity and of entropy smaller than those observed without inhibitors, indicating that inhibitors increase the monomer order. The temperature dependence of the equilibrium dissociation constant $K_D$ is reported, for each inhibitor, in Supplementary Fig. S1. The large uncertainties on the fitting parameters determines the presence of wide bands of uncertainty on the $K_D$ trends. This is particularly evident for inhibitor **5**, since SAXS data have been recorded only for one inhibitor concentration. Hence, a word of caution is necessary regarding the temperature dependence of $K_D$ in the presence of the seven inhibitors obtained by the SAXS analysis.

From a close inspection of the single curve parameters, reported in Supplementary Tables S1 and S2, we observe that the value of the correlation length $\xi$ is in the range 3000-4500 Åand rather independent of the temperature and the presence of the inhibitors. The fractal dimension is $\approx 2$, suggesting a two-dimensional fractal growth of protein clusters in the presence of inhibitors.

## Discussion

The active site of M^pro^ monomer, which is highly conserved in different coronaviruses, is typically composed of four subsites, referred to as S1$^\prime$, S1, S2, and S4^41–43^. They accommodate the corresponding domains P1$^\prime$, P1, P2, and P4 of the substrate or the ones of the inhibitor compound mimicking the substrate^44^. The S1$^\prime$ subsite is constituted by the two residues Thr24 and Thr25. The S1 subsite (also referred to as the S1 pocket^43^) is formed by the side chains of residues Phe140, Asn142, Glu166, His163 and His172 and by the main chains of Phe140 and Leu141^44^. As discussed by Sacco et al.^43^, S1 is considered a promising target for an inhibiting compound, as it can interact with both hydrophobic and hydrophilic groups. On the other hand, S2 is a hydrophobic subsite formed of the side chains of His41, Met49, Tyr54, Met165 and Asp187, while S4 is a small hydrophobic pocked that involves the side chains of Met165, Leu167, Phe185, Gln192 and Gln189^44^. An unusual catalytic dyad, His41-Cys145, acts in the active site, where His41 is a proton acceptor whereas Cys145 is attacked by the carbonyl carbon of the substrate. Hence, a signature of the inhibiting power of a compound is its capability to form a covalent bond with Cys145^41^, as very recently confirmed by Dai et al.^42^, who have found two promising inhibitors **11a** and **11b**. The importance of the protonation state of Cys145 as well as the network of hydrogen bonds between the catalytic site of M^pro^ and inhibiting compounds has also been recently discussed by Kneller et al.^45^ by combining X-ray and neutron scattering data. On these grounds, the experimental results obtained in the present study, together with the structure of the seven inhibitors within the M^pro^ active site determined by the refined molecular docking, can be discussed.

The interaction map of inhibitor **1** is shown in Supplementary Fig. S5. There are a total of 11 contacts with amino acids of M^pro^ monomer (Ser1, Thr25, Thr26, Ser46, Asn119, Leu141, Asn142, Cys145, Pro168, Arg188, Gln192), 2 of them (Ser46, Asn119) are hydrogen bonds. The residues of the catalytic dyad and the four subsites in contact with **1** are: Cys145 (dyad, 1 of 2 (50 of 2 (50 5 (20 To note, these contacts involve only one of the residues of the catalytic dyad, Cys145, without a hydrogen bond, whereas for inhibitor **13b** there is a hydrogen bond with Cys145 (Supplementary Fig. S3). It is also worth to notice that Ser1 is among the residues in contact with inhibitor **1**: since the mutual interaction of Glu166 of one monomer and the N-finger residues of the other monomer, like Ser1, has been proven to shape the catalytic cleft^7^, we argue that this compound could destabilize the dimer, consistently with the high value of $K_D^\circ =26 \pm 4$ $\mu$M at $C_{\mathrm{I}}=60$ $\mu$M. However, its enzymatic inhibition is very poor, as shown by the high similarity of the RFU slope with the one in the absence of inhibitors (Fig. 5). A possible explanation of this result could be the absence of any hydrogen bond with Cys145 as well as the absence of any contact with the residues of subsite S2.

Regarding inhibitor **2**, the map of contacts shown in Supplementary Fig. S6 reveals a total of 10 interactions with the monomer chain (Met49, Asn142, Gly143, Cys145, Asp187, His164, Met165, Glu166, Arg188, Gln189); 4 of them are hydrogen bonds (Asn142, His164, Glu166, Gln189) that do not involve the catalytic site. More in detail, the residues of the catalytic dyad and the four subsites in contact with **2** are: Cys145 (dyad, 1 of 2 (50 Asn142 and Glu166 (S1, 2 of 6 (33 of 5 (60 We also note that 2 residues of S1, Asn142 and Glu166, interact with this inhibitor via a hydrogen bond. This evidence, together with the high number of contacts with S2 and S4, could explain the experimentally observed inhibition effect ($m=5 \pm 2$ RFU/min, Fig. 5). To note, this compound does not modify the dimer-monomer equilibrium, being the fitting parameter $\alpha _G$ almost 0 (Table 3) within the experimental error.

Turning now to inhibitor **3**, we have found that it does not alter the native dissociation equilibrium of M^pro^ ($\alpha _G\approx 0$, see Table 3) and also its inhibition effect, observed by fluorescence analysis, is week ($m=23\pm 3$ RFU/min, slightly lower that the value in absence of inhibitors). The contact map of compound **3** (Fig. 4) shows a total of 12 interactions with the monomer chain (Thr24, Thr25, Thr26, His41, Ser46, Met49, Phe140, Leu141, Gly143, Met165, Leu167, Gln192), 2 of them being hydrogen bonds (Thr24, Ser46) and one (His41) a $\pi -\pi$ stacking. The residues of the catalytic dyad and the four subsites in contact with **3** are: His41 (dyad, 1 of 2 (50 (S1$^\prime$, 2 of 2 (100 His41, Met49 and Met165 (S2, 3 of 5 (60 (S4, 3 of 5 (60 We notice that with respect to compound **2**, there are no hydrogen bonds with the five residues that stabilize the S1 pocket. This difference might be the reason for the weak inhibition effect.

Compound **4** is the most effective among the seven inhibitors ($m\approx 0$, Fig. 5) even if it shows at $C_{\mathrm{I}}=30$ $\mu$M a dissociation equilibrium constant $K_D^\circ =6 \pm 2$ $\mu$M slightly lower than the one without inhibitors (see Table 1). Indeed the parameter $\alpha _G$ is positive but very close to 0 within the experimental error (Table 3), suggesting that compound **4** provokes a very weak stabilizing effect of the dimer. The contact map (Supplementary Fig. S7) shows 11 interactions with the monomer chain (Thr25, Thr26, Leu27, His41, Ser46, Met165, Glu166, Pro168, Gln189, Thr190, Gln192), including one hydrogen bond (Gln189). The residues of the catalytic dyad and the four subsites in contact with **4** are: His41 (dyad, 1 of 2 (50 of 2 (50 (40 Only one of the five residues that stabilize the S1 pocket is among the ones in contact with this inhibitor, Glu166, which does not form a hydrogen bond. On this ground, the high inhibition effect of compound **4** could be only justified by the contact with His41, one of the two residues of the catalytic dyad.

Results are different for compound **5**: at $C_{\mathrm{I}}=60$ $\mu$M (unfortunately no data are available at 30 $\mu$M) it provokes a rather important increase of $K_D^\circ$ (Table 3) and shows a moderate inhibition effect ($m=8 \pm 3$ RFU/min, see Fig. 5). Looking at the interaction map (Supplementary Fig. S8), we notice 11 interactions with monomer M^pro^ (Thr25, Thr26, Leu27, His41, Asn142, Gly143, Met165, Glu166, Pro168, Arg188, Gln192), one of them regards Glu166, involved in two hydrogen bonds, and the other one His41, involved in two a $\pi -\pi$ stacking interactions. The residues of the catalytic dyad and the four subsites in contact with **5** are: His41 (dyad, 1 of 2 (50 of 2 (50 Met165 (S2, 2 of 5 (40 There are not hydrogen bonds involving the five residues that stabilize the S1 site. One could speculate that this inhibitor, probably due to its steric hindrance, provokes a modification of S1 that could interfere with the enzymatic activity of M^pro^.

Compound **6** determines 11 contacts with the amino acid of the monomer (His41, Leu141, Ser144, Cys145, Met165, Glu166, Leu167, Arg188, Gln189, Ala191, Gln192, Supplementary Fig. S9), including two hydrogen bond (His41, Gln192). In particular, the residues of the catalytic dyad and the four subsites in contact with **6** are: His41 and Cys145 (dyad, 2 of 2 (100 2 of 5 (40 An almost absent inhibition effect is seen by fluorescence, being the slope of RFU (Fig. 5) very similar to the one determined in the absence of inhibitors. On the other side, compound **6** is able to modify the dimer-monomer dissociation, with one of the highest values of $K_D^\circ =30\pm 10$ $\mu$M at $C_{\mathrm{I}}=60$ $\mu$M (Table 3). To note, only one of the 6 amino acids that stabilize the S1 site are included in the list of residues interacting with compound **6**. Hence, the absence of its inhibition activity could be explained by the small size of its molecular structure, which might not be able to provoke important modifications of the S1 pocket and hence to modify the catalytic features of M^pro^.

We finally turn to compound **7**. It shows an opposite behaviour with respect to compound **6**: it is capable to change the dimer-monomer equilibrium at the same extent ($K_D^\circ =30\pm 10$ $\mu$M at $C_{\mathrm{I}}=60$ $\mu$M, Table 3) and displays a promising inhibition effect, with $m \approx 1$. For this compound, the map of contacts shows 12 interactions (Thr24, Thr25, Thr26, His41, Thr45, Met49, Leu141, His164, Met165, Glu166, Asp187, Arg188, Fig. 4) with a large number of hydrogen bonds (Thr24, Thr26, His41, His164, Glu166, Arg188). The residues of the catalytic dyad and the four subsites in contact with **7** are: His41 (dyad, 1 of 2 (50 (S1$^\prime$, 2 of 2 (100 His41, Met49, Met165 and Asp187 (S2, 4 of 5 (80 5 (20 Only one of the interacting residues (Glu166) is involved in the stabilization of the S1 pocket. We can infer the high inhibition effect could be due to the high number of contact with S2 and to the presence of 6 hydrogen bonds. Another hypothesis, which needs further insights to be confirmed, is that the fluorinated groups, which are present in a high number in compound **7**, may originate a new reactive warhead able to form a covalent bond with Cys145. We may also consider that one of them involves a residue of the catalytic dyad, His41, suggesting a possible important modification of the enzymatic activity.

## Conclusions

Considering the results that we have discussed above, a picture emerges where the selected compounds designed to bind the catalytic site of SARS-CoV-2 M^pro^ may affect dimerization and enzymatic activity processes to a different extent. Since the functional form of M^pro^ is a dimer, compounds that disrupt dimerization should be in principle also effective at diminishing its catalytic activity. However, the compound-induced shift in the dimer and monomer thermal equilibrium populations may not directly translate into a loss of enzymatic activity. Indeed, the latter strongly depends also on the local interactions occurring at the catalytic site, that in turn governs the competition between inhibitor and substrate. To better visualize the scenario presented by our findings, we report in Fig. 6 (right panel) the slope *m* of the fluorescence inhibition curve as a function of the dimer-monomer equilibrium constant $K_D$ calculated from the set of fitting parameters, derived by the global fit of SAXS data of the seven compounds, by fixing the inhibitor concentration $C_{\mathrm{I}}$ at 60 $\mu$M and the temperature at $30^\circ$ C, the same value employed in the enzymatic activity assays. The points in this map could be organized in two groups, as represented in blue and in red. The latter group refers to compounds **3**, **5** and **7**, which show the expected behaviour: the stronger is their capability to induce the dissociation of the M^pro^ dimer, the more important is their inhibiting effect. For these compounds the molecular mechanisms underlying inhibition at the active site are likely linked with their ability to provoke dimer dissociation. On the other hand, for compounds **1**, **2**, **4**, and **6**, displayed in blue, we find the opposite behavior: the increase of dimer dissociation does not determine an increase of inhibition, thus suggesting that the molecular mechanisms of inhibition at the active site play a major role with a marginal involvement of the monomer-monomer interface. This apparently contradictory result can be in part explained by considering that, in all cases, the dissociation equilibrium is weak so that, in the presence of a compound that alters the dimer-monomer equilibrium but that does not hamper the interaction with the substrate, there are always dimeric M^pro^ molecules that can exert their enzymatic activity when a substrate is available.

A further complementary explanation could be the existence of ligand binding sites alternative to the orthosteric active site located at the interface of dimeric M^pro^. Indeed, the presence of two of such binding sites, not directly involved in enzymatic inhibition but probably interfering with dimerization, has been very recently revealed by a molecular dynamics simulation study in the SARS-CoV-2 M^pro^^46^.

We can provide a qualitative interpretation of the behavior exhibited by the two groups of compounds by looking at the contact maps of the residues grouped in the different sub-sites of the active site. We note that both compound **6** and **7**, which show the higher values of $K_D$, are in contact with two residues of the S1, Leu141 and Glu166. Besides, compound **1**, which has a slightly lower $K_D$, interacts with S1 through the contacts with Leu141 and Asn142. This suggests that binding with at least two of the residues Glu166, Leu141 and Asn142 is crucial to modify the dimer-monomer equilibrium. On the other hand, the interaction of compounds **7**, **2** and **5** with Glu166 via an hydrogen bond is likely linked to their high inhibiting action. On the contrary, there is no hydrogen bond between compound **6** and Glu166. Hence, on the basis of the results obtained for the seven selected compounds, the interplay between SAXS results, enzymatic activity assays and contact map analysis suggests a relevant clue: in order to promote both M^pro^ dimer dissociation and the inhibition of its catalytic activity, a small molecule should interact with at least two residues of the S1 sub-site and most likely form an hydrogen bond with Glu166. The key role of Glu166 residue, which is conserved among all human coronaviruses, for inhibition has been pointed out also very recently^47^.

To note, according to Goyal and Goyal^6^, Glu166 is among the residues that should be targeted to inhibit the dimerization of SARS-CoV M^pro^. However, for a more detailed investigation of the dimerization process in stabilizing the catalytic activity of M^pro^, it is also important to take into account the overall contribution of protein flexibility, as recently evidenced by Suárez et al.^48^, through a 2 $\mu$s Molecular Dynamics simulation of M^pro^ with and without a model peptide mimicking the enzyme substrate.

In summary, the experimental work presented here brings basic information to decipher the complex interplay between enzymatic activity inhibition and dimer dissociation. To the best of our knowledge, we have shown for the first time how structural information about the SARS-CoV-2 M^pro^ in solution in the absence and in the presence of potential inhibitors and as a function of temperature can be obtained from an advanced analysis of SAXS data within an overall thermodynamic picture, complemented by more conventional approaches. Our results suggest that more experimental evidences about the impairment of monomer and dimer M^pro^ in the presence of inhibitors corroborated by computational information will be necessary for a deeper understanding of the M^pro^ allosteric mechanism.

## Materials and methods

### M^pro^ expression and western blot analysis

pGEX-6P-1 vector harboring the full length cDNA sequence encoding for SARS-CoV-2 Main Protease (M^pro^ NC_045512) was purchased from GenScript (clone ID_M16788F). The expressing vector was transformed into BL21DE3pLys *Escherichia coli* cells and the obtained clones were assayed both in small scale (5 mL) and medium scale (500 mL and 1 L) for the production of SARS-CoV-2 M^pro^. Transformants were grown onto LB medium containing 100 $\mu$g/mL Ampicillin and 34 $\mu$g/mL Chloramphenicol as selective antibiotics. Cultures were grown up to OD600 of 0.6-0.8 at 37$^\circ$ C, 200 rpm and then M^pro^ expression was induced by addition of 0.5 mM isopropyl-1-thio-$\beta$-D-galactopyranoside (IPTG). Growth under induction was achieved both for 3 h at 37$^\circ$ C and 10 h at 16$^\circ$ C in order to test the best expressing condition. Cells were harvested by centrifugation at 6000 g. Cell pellets were resuspended in lysis buffer (20 mM Tris-HCl pH 8.0, 300 mM NaCl, 2 mM $\beta$-mercaptoethanol), and cell rupture was achieved by sonication (Sonics Vibra Cell sonicator) at 4$^\circ$ C. Cell debris was separated from the total protein extract by centrifugation at 6500 g for 1 h. Supernatant aliquotes were resuspended in Laemmli sample buffer, run onto 12 polyvinylidene difluoride membrane for Western blot analysis. M^pro^ was decorated by 6$\times$-His tag monoclonal primary antibody (Invitrogen) and anti-mouse secondary antibody and detected by chemiluminescence (Clarity Western ICL Substrate, Biorad, Supplementary Fig. S11, panels A and B).

### M^pro^ purification and His-tag cleavage

The total cell extract was loaded onto Ni-NTA affinity column (G-Biosciences) and washed by washing buffer (Tris-HCl 20 mM pH 7.6, NaCl 100 mM). M^pro^ was eluted by elution buffer (Tris-HCl 20 mM pH 7.6, NaCl 100 mM, 300 mM imidazole) in 5 fractions of 1 mL each. Aliquotes of elution fractions were loaded onto 12 acrylamide gel and imidazole was removed by dialysis against Prescission cleavage buffer (Tris-HCl 50 mM pH 8.0, NaCl 150 mM, dithiothreitol 1 mM, ethylenediaminetetraacetic acid 1 mM) through Amicon Ultra-4 centrifugal filters 30K (Merck Millipore). For M^pro^ C-terminal His-tag removal, the Prescission (1 U for 100 $\mu$g of protein) cleavage reaction was performed at 4$^\circ$ C for 4 h and Prescission protease was then removed by GSTrap FF column (GE-Healthcare). The M^pro^ solution was further purified by FPLC size-exclusion chromatography on Superdex 75 10/300 GL column (Supplementary Fig. S10, panels A and B)^33,44^.

### M^pro^ activity assay and inhibition

The fluorescently labelled auto-cleavage sequence of SARS-CoV-2 M^pro^, ((7-Methoxycoumarin-4-yl)acetyl)-AVLQ$\downarrow$SGFRK(2,4-dinitrophenyl)K (purchased from GenScript), was utilized to monitor the recombinant M^pro^ kinetics (excitation 320 nm, emission 405 nm). The assay was started by mixing $\approx 0.2$ $\mu$M SARS-CoV-2 M^pro^ to different amounts of substrate (10, 20, 40 $\mu$M) in order to set the best protein-substrate concentration to detect M^pro^ activity^41^. Fluorescence intensity was measured by DeNovix DS-11 FX+ fluorometer. The M^pro^ activity reported as reference for inhibition tests was obtained by linear fitting of the fluorescence curve in the presence of 40 $\mu$M of substrate concentration^33,41^. Seven inhibitors dissolved in dimethyl sulfoxide (DMSO) were tested at a final concentration of 30 $\mu$M^42^ (Fig. 5). Each reaction in a final volume of 200 $\mu$L was firstly incubated for 20 min at 30$^\circ$ C without substrate. After substrate addition, fluorescence intensities were reported as relative fluorescence units (RFU) and monitored every minute for a duration of 30 min at 30$^\circ$ C.

### Circular dichroism

In-house circular dichroism experiments were performed at room temperature using a JASCO J-810 spectropolarimeter (Physics and Geology Department, University of Perugia). Quartz cuvettes with path-length of 1 mm was used, in order to obtain the optimum signal-to-noise ratio for the M^pro^ samples with concentrations of 16, 6 and 2.5 $\mu$M respectively. Protein concentration was measured by performing absorption measurements on the same samples, with an extinction coefficient of 33640 $\hbox {M}^{-1}$ $\hbox {cm}^{-1}$ estimated from amino acid sequence Expasy online ProtParam tool^49^. Each spectrum was collected in the range from 200 to 260 nm with a scan speed of 50 nm/min, and repeated three times. The CD data are represented in molar extinction units, by using the formula $[\Theta ]=m^\circ /(10 \,C \,L)$, where $m^\circ$ is the ellipticity (in millidegree unit), *C* is the protein molar concentration and *L* is the path length of cell (in cm). The thermal stability has been studied at 16 $\mu$M M^pro^ concentration, by varying the temperature through a thermal bath from $27^\circ$ to $77^\circ$ C.

### Small angle X-ray scattering

SAXS experiments were carried out at the B21 beam-line of the Diamond Synchrotron (Didctot, UK), operating with a fixed camera length (4.014 m) at 12.4 keV ($\lambda =1.000$ Å) and with a flux of $\sim 10^{12}$ photons per second. Samples were injected in the capillary (thickness 1.7 mm) by means of a robotic apparatus and measured 21 times with an exposure time of 1 min. The M^pro^ samples without inhibitors were measured at the nominal monomer molar concentration of 3, 10, 20 and 30 $\mu$M and at temperature of 15$^\circ$, 25$^\circ$, 30$^\circ$, 37$^\circ$ and 45$^\circ$ C. In the presence of inhibitors, SAXS curves were recorded at two M^pro^ monomer molar concentrations, 30 and 60 $\mu$M, and at three temperatures, 30$^\circ$, 37$^\circ$ and 45$^\circ$ C.

SAXS data analysis approach has been described in the main text, with the exception of some minor points. Since in all conditions the nominal molar protein concentration is lower than 1 mM, its temperature variations can be considered to be only determined by the dependency with *T* of the relative mass density of water, which, according to literature results^50^ is written as

9 $$\begin{aligned} d_{\mathrm{w}}= & {} e^{-\alpha _{\mathrm{w}}(T-T_\circ )-\beta _{\mathrm{w}}(T-T_\circ )^2/2}, \end{aligned}$$

where, in our investigated range $15-45^\circ$ C, the optimum value of the thermal expansivity at $T_\circ$ is $\alpha _{\mathrm{w}}=2.5 \cdot 10^{-4}$ $\hbox {K}^{-1}$ and the one of its first derivative is $\beta _{\mathrm{w}}=9.8 \cdot 10^{-6}$ $\hbox {K}^{-2}$. Accordingly, $C_N=C_\circ d_{\mathrm{w}}$, $C_\circ$ being the nominal protein concentration at $T_\circ$.

The measured structure factor $S_M(q)$ has been obtained in relation to the protein-protein structure factor *S*(*q*) by:

10 $$\begin{aligned} S_M(q)=1+\beta (q)[S(q)-1] \end{aligned}$$

where $\beta (q)$ is the coupling function

11 $$\begin{aligned} \beta (q)= & {} \frac{|P^{(1)}(q)|^2}{P(q)} \end{aligned}$$

and $P^{(1)}(q)$ is the average of the protein excess scattering amplitude, a function provided, together with *P*(*q*) by the SASMOL method. According to Ref.^24^, *S*(*q*) has been written as

12 $$\begin{aligned} S(q)=1+\frac{1}{(qr_0 )^D} \frac{D\Gamma (D-1)}{[1+(q\xi )^{-2}]^{D(D-1)/2} }\sin [(D-1)\tan ^{-1}(q\xi )], \end{aligned}$$

where $\Gamma (x)$ is the gamma function, *D* is the fractal dimension (comprised between 1 and 3) of the aggregates, $r_0$ is the effective radius of the aggregating protein and $\xi$ is the correlation length.

### In-silico design

#### Ligand preparation

The default setting of the LigPrep tool implemented in Schrödinger’s software (version 2017-1) was used to prepare the ligands for docking^51^. All possible tautomers and combination of stereoisomers were generated for pH $7.0 \pm 0.4$, using the Epik ionization method^52^. Energy minimization was subsequently performed using the integrated OPLS 2005 force field^53^.

#### Protein preparation

The crystal structure of SARS-CoV-2 M^pro^ in complex with ligand **13b** (PDB code 6y2f)^7^ was downloaded from the Protein Data Bank^54^. The cocrystal ligand, covalently bonded to Cys145, was treated by breaking the covalent bond and filling in open valence. Protein Preparation Wizard of Schrödinger software was subsequently employed for further preparations of the protein structure using the default settings^55^. Bond orders were assigned, and hydrogen atoms as well as protonation of the heteroatom states were added using the Epik-tool (with the pH set at biologically relevant values, i.e. at $7.0 \pm 0.4$). The H-bond network was then optimized. The structure was subjected to a restrained energy minimization step (RMSD of the atom displacement for terminating the minimization was 0.3 Å), using the Optimized Potentials for Liquid Simulations (OPLS) 2005 force field^53^.

#### Docking validation

Molecular Docking was performed by the Glide program^37,56,57^. The receptor grid preparation was performed by assigning the original ligand (**13b**) as the centroid of the grid box. The generated 3D conformers were docked into the receptor model using the Standard Precision (XP) mode as the scoring function. A total of 5 poses per ligand conformer were included in the post-docking minimization step, and a maximum of 2 docking poses were generated for each ligand conformer. The proposed docking procedure was validated by the re-dock of the crystallized **13b** within the receptor-binding pockets of 6y2f by Glide covalent docking. The results obtained were in good agreement of the experimental poses, showing a RMSD of 0.75 Å.

#### Biotarget finder module (DRUDIT)

The refined selection of suitable SARS-CoV-2 M^pro^ inhibitors was performed through the module Biotarget Finder as available in the www.drudit.com webserver^36^. The tool allows to predict the binding affinity of candidate molecules versus the selected biological target. The template of the biological target was built as previously reported. Thus, the in-house database was submitted to the Biological Predictor module by setting the DRUDIT parameters, *N*, *Z*, and *G*, using the crystallized structure of **13b**, as previously reported^35^.

#### Induced fit docking

Induced fit docking simulation was performed using the IFD application as available^38,58^ in the Schrödinger software suite^39^, which has been demonstrated to be an accurate and robust method to account for both ligand and receptor flexibility^59^. The IFD protocol was performed as follows^60,61^: the ligands were docked into the rigid receptor models with scaled down van der Waals (vdW) radii. The Glide Standard Precision (XP) mode was used for the docking and 20 ligand poses were retained for protein structural refinements. The docking boxes were defined to include all amino acid residues within the dimensions of 25 Å$\times$25 Å$\times$25 Å from the centre of the original ligands. The induced-fit protein-ligand complexes were generated using Prime software^39,62,63^. The 20 structures from the previous step were submitted to side chain and backbone refinements. All residues with at least one atom located within 5.0 Å of each corresponding ligand pose were included in the refinement by Prime. All the poses generated were then hierarchically classified, refined and further minimized into the active site grid before being finally scored using the proprietary GlideScore function defined as follows: $\mathrm{XPG\_score}= 0.065\, \mathrm{vdW} + 0.130\,\mathrm{Coul} + \mathrm{Lipo} + \mathrm{Hbond} + \mathrm{Metal} + \mathrm{BuryP} + \mathrm{RotB} + \mathrm{Site}$, where ${\mathrm{vdW}}$ is the van der Waals energy term, ${\mathrm{Coul}}$ is the Coulomb energy, ${\mathrm{Lipo}}$ is a lipophilic contact term that rewards favourable hydrophobic interactions, ${\mathrm{Hbond}}$ is an H-bonding term, ${\mathrm{Metal}}$ is a metal-binding term (where applicable), ${\mathrm{BuryP}}$ is a penalty term applied to buried polar groups, ${\mathrm{RotB}}$ is a penalty for freezing rotatable bonds and ${\mathrm{Site}}$ is a term used to describe favourable polar interactions in the active site. Finally, ${\mathrm{IFD\_score}}$ (${\mathrm{IFD\_score}} = {\mathrm{XPG\_score}} + 0.05\, {\mathrm{Prime\_Energy}}$), which accounts for both protein-ligand interaction energy and total energy of the system, was calculated and used to rank the IFD poses. More negative ${\mathrm{IFD\_score}}$ values indicated more favourable binding. Results are shown in Table 2.

### Chemical synthesis of inhibitors

Inhibitors **1**^64^, **3**^64^, **5**^64^ and **6**^65^ have been prepared as previously reported. Inhibitor **2** is commercial. Inhibitors **4**^64^ and **7**^64^ have been synthesized as described in detail in the next paragraphs. All solvent and reagents were used as received, unless otherwise stated. Melting points were determined on a hot-stage apparatus. $^1$H-NMR and $^{13}$C-NMR spectra were recorded at indicated frequencies, residual solvent peak was used as reference. Chromatography was performed by using silica gel (0.040-0.063 mm) and mixtures of ethyl acetate and petroleum ether (fraction boiling in the range of 40-60$^\circ$ C) in various ratios (v/v). Compounds **8**^64^ and **9**^66^, used in the synthesis of inhibitors **4** and **7**, have been prepared as previously reported.

#### Synthesis of inhibitor **4**

Inhibitor **4** was synthesized through a nucleophilic aromatic substitution (S_N_Ar) of 5-pentafluorophenyl-1,2,4-oxadiazole **8** with 1-Aza-18-crown-6 in para position (Supplementary Fig. S12). Oxadiazole **8** (312 mg, 1 mmol) was dissolved in acetonitrile (5 mL). 1-Aza-18-crown-6 (289 mg, 1.1 mmol) and potassium carbonate (152 mg, 1.1 mmol) were added and the suspension was stirred at room temperature for 24 h. The reaction was monitored by TLC. The reaction mixture was dried under vacuum and treated with $\hbox {H}_2$O (50 mL) before extraction three times with EtOAc (50 mL each). The combined organic layers were dried with $\hbox {Na}_2\hbox {SO}_4$ and then concentrated in vacuo to give the crude product, which was recrystallized from EtOH. **16-(2,3,5,6-tetrafluoro-4-(3-phenyl-1,2,4-oxadiazol-5-yl)phenyl)-1,4,7,10,13-pentaoxa-16-azacyclooctadecane**: yield: 73 $\delta$: 3.68-3.80 (m, 24H, overlapped $-\hbox {CH}_2-$ signals), 7.51-7.54 (m, 3H, Ar), 8.16-8.20 (m, 3H, Ar). FTIR (Nujol) 1647, 1529, 1518 $\hbox {cm}^{-1}$; HRMS-ESI [(M+H)$^+$]: *m/z* calculated for ($\hbox {C}_{{26}}\hbox {H}_{{30}}\hbox {F}_4\hbox {N}_2\hbox {O}_{{6}}$)$^+$: 556.2060; found, 556.2046.

#### Synthesis of inhibitor **7**

Tripodal oxadiazolylamide (inhibitor **7**) was easily obtained by means of nucleophilic displacement with ethylamine from tripodal ester **9**, which was previously reported as heavy metal fluorescent sensor (Supplementary Fig. S13)^66^. Tripodal **9** (101 mg, 0.1 mmol) was dissolved in acetonitrile (3 mL). Ethylamine (2 M in MeOH, 150 $\mu$L, 0.3 mmol) was added and the solution was stirred at room temperature for 24 h. The reaction was monitored by TLC. The reaction mixture was dried under vacuum and treated with $\hbox {H}_2$O (50 mL) before extraction three times with EtOAc (50 mL each). The combined organic layers were dried with $\hbox {Na}_2\hbox {SO}_4$ and then concentrated in vacuo to give the crude product, which was purified by chromatography. **5,5’,5”-(((nitrilotris(ethane-2,1-diyl))tris(azanediyl))tris(2,3,5,6-tetrafluorobenzene-4,1-diyl))tris(N-ethyl-1,2,4-oxadiazole-3-carboxamide)**: yield: 66 d $_6$) $\delta$: 1.18 (t, 9H, $J= 6.9$ Hz, $\hbox {CH}_3$), 2.85 (bs, 6H, $\hbox {NCH}_2\hbox {CH}_2$NH-), 3.34-3.48 (m, 6H, $\hbox {CH}_3\hbox {CH}_2$NH-), 3.56 (bs, 6H, $\hbox {NCH}_2\hbox {CH}_2$NH-), 6.91 (bs, 3H, $\hbox {NCH}_2\hbox {CH}_2$NH-), 9.02 (t, 3H, $J= 5.7$ Hz, $\hbox {NHCH}_2\hbox {CH}_3$). FTIR (Nujol) 3325, 1701, 1680, 1647 $\hbox {cm}^{-1}$; HRMS-ESI [(M+H)$^+$]: *m/z* calculated for ($\hbox {C}_{{39}}\hbox {H}_{{34}}\hbox {F}_{{12}}\hbox {N}_{{13}}\hbox {O}_{{6}}$)$^+$: 1007.2485; found, 1007.2518.

## Supplementary information

Supplementary information.

## References

[1] Wang C, Horby PW, Hayden FG, Gao GF (2020). A novel coronavirus outbreak of global health concern. Lancet.

[2] Zhou, P. *et al.* A pneumonia outbreak associated with a new coronavirus of probable bat origin. *Nature* **579**, 270+, 10.1038/s41586-020-2012-7 (2020). 10.1038/s41586-020-2012-7 PMC7095418 32015507

[3] Anand K, Ziebuhr J, Wadhwani P, Mesters J, Hilgenfeld R (2003). Coronavirus main proteinase (3CL(pro)) structure: basis for design of anti-SARS drugs. Science.

[4] Morse JS, Lalonde T, Xu S, Liu WR (2020). Learning from the past: possible urgent prevention and treatment options for severe acute respiratory infections caused by 2019-nCoV. ChemBioChem.

[5] Zhang D (2010). Evolutionary selection associated with the multi-function of overlapping genes in the hepatitis B virus. Infect. Genet. Evol..

[6] Goyal B, Goyal D (2020). Targeting the dimerization of the main protease of coronaviruses: a potential broad-spectrum therapeutic strategy. ACS Comb. Sci..

[7] Zhang L (2020). Crystal structure of SARS-CoV-2 main protease provides a basis for design of improved alpha-ketoamide inhibitors. Science.

[8] Shi J, Song J (2006). The catalysis of the SARS 3C-like protease is under extensive regulation by its extra domain. FEBS J..

[9] Anand K (2002). Structure of coronavirus main proteinase reveals combination of a chymotrypsin fold with an extra alpha-helical domain. Embo J..

[10] Chen S (2005). Severe acute respiratory syndrome coronavirus 3C-like proteinase n terminus is indispensable for proteolytic activity but not for enzyme dimerization: biochemical and thermodynamic investigation in conjunction with molecular dynamics simulations. J. Biol. Chem..

[11] Chou C (2004). Quaternary structure of the severe acute respiratory syndrome (SARS) coronavirus main protease. Biochemistry.

[12] Spinozzi F, Gazzillo D, Giacometti A, Mariani P, Carsughi F (2002). Interaction of proteins in solution from small angle scattering: a perturbative approach. Biophys. J..

[13] Gottschalk M, Nilsson H, Roos H, Halle B (2003). Protein self-association in solution: the bovine $$\beta $$-lactoglobulin dimer and octamer. Protein Sci..

[14] Ortore MG (2005). High pressure small-angle neutron scattering study of the aggregation state of $$\beta $$-lactoglobulin in water and water/ethylene glycol solutions. Chem. Phys. Lett..

[15] Russo, D. *et al.* The impact of high hydrostatic pressure on structure and dynamics of beta-lactoglobulin. *Biochimica et Biophysica Acta (BBA)-General Subjects* **1830**(10), 4974–4980 (2013). 10.1016/j.bbagen.2013.06.040 23850562

[16] Molodenskiy D (2017). Thermally induced conformational changes and protein-protein interactions of bovine serum albumin in aqueous solution under different ph and ionic strengths as revealed by saxs measurements. Phys. Chem. Chem. Phys..

[17] Sauter A (2016). Structural evolution of metastable protein aggregates in the presence of trivalent salt studied by (v)sans and saxs. J. Phys. Chem. B.

[18] Salter JD, Krucinska J, Raina J, Smith HC, Wedekind JE (2009). A hydrodynamic analysis of apobec3g reveals a monomer-dimer-tetramer self-association that has implications for anti-hiv function. Biochemistry.

[19] Deyaert E (2017). A homologue of the Parkinson’s disease-associated protein LRRK2 undergoes a monomer-dimer transition during GTP turnover. Nat. Commun..

[20] Graziano V, McGrath WJ, Yang L, Mangel WF (2006). SARS CoV main proteinase: the monomer-dimer equilibrium dissociation constant. Biochemistry.

[21] Spinozzi, F. Genfit, version 2020. (2020).

[22] Spinozzi F, Ferrero C, Ortore MG, Antolinos ADM, Mariani P (2014). GENFIT: software for the analysis of small-angle X-ray and neutron scattering data of macromolecules in-solution. J. App. Cryst..

[23] Ortore MG (2009). Combining structure and dynamics: non-denaturing high-pressure effect on lysozyme in solution. J. R. Soc. Interface.

[24] Teixeira J (1988). Small-angle scattering by fractal systems. J. Appl. Cryst..

[25] Apenten, R., Khokhar, S. & Galani, D. Stability parameters for $$\beta $$-lactoglobulin thermal dissociation and unfolding in phosphate buffer at pH 7.0. *Food Hydrocolloids* **16**, 95 – 103 (2002).

[26] Janin, J. Elusive affinities. *Proteins: Struct. Funct. Bioinform.* **21**, 30–39 (1995). 10.1002/prot.340210105 7716167

[27] Horton N, Lewis M (1992). Calculation of the free energy of association for protein complexes. Protein Sci..

[28] Svergun D (1998). Protein hydration in solution: experimental observation by X-ray, neutron scattering. Proc. Natl. Acad. Sci. USA.

[29] Sinibaldi R (2007). Preferential hydration of lysozyme in water/glycerol mixtures: a small-angle neutron scattering study. J. Chem. Phys..

[30] Sinibaldi R (2008). Sans/saxs study of the bsa solvation properties in aqueous urea solutions via a global fit approach. Eur. Biophys. J..

[31] Greenfield NJ, Fasman GD (1969). Computed circular dichroism spectra for the evaluation of protein conformation. Biochemistry.

[32] Greenfield NJ (2006). Using circular dichroism collected as a function of temperature to determine the thermodynamics of protein unfolding and binding interactions. Nature Protocols.

[33] Abian O (2020). Structural stability of SARS-CoV-2 3CLpro and identification of quercetin as an inhibitor by experimental screening. Int. J. Biol. Macromol..

[34] Shi J, Wei Z, Song J (2004). Dissection study on the severe acute respiratory syndrome 3C-like protease reveals the critical role of the extra domain in dimerization of the enzyme: defining the extra domain as a new target for design of highly specific protease inhibitors. J. Biol. Chem..

[35] Martorana A, Gentile C, Lauria A (2020). In silico insights into the SARS CoV-2 main protease suggest NADH endogenous defences in the control of the pandemic coronavirus infection. Viruses.

[36] Lauria A (2019). DRUDIT: web-based DRUgs DIscovery Tools to design small molecules as modulators of biological targets. Bioinformatics.

[37] Friesner, R. A. *et al.* Glide: a new approach for rapid, accurate docking and scoring. 1. Method and assessment of docking accuracy. *J. Med. Chem.* **47**, 1739–1749 (2004). 10.1021/jm0306430 15027865

[38] Sherman W, Day T, Jacobson MP, Friesner RA, Farid R (2006). Novel procedure for modeling ligand/receptor induced fit effects. J. Med. Chem..

[39] Schrödinger, N. Y., LLC. Maestro, version 10.2 (2017).

[40] Lee, J. *et al.* Crystallographic structure of wild-type SARS-CoV-2 main protease acyl-enzyme intermediate with physiological C-terminal autoprocessing site. *NATURE COMMUNICATIONS* **11**, 10.1038/s41467-020-19662-4 (2020). 10.1038/s41467-020-19662-4 PMC7674412 33208735

[41] Yang H (2005). Design of wide-spectrum inhibitors targeting coronavirus main proteases. PLOS Biol..

[42] Dai W (2020). Structure-based design of antiviral drug candidates targeting the sars-cov-2 main protease. Science.

[43] Sacco, M. D. *et al.* Structure and inhibition of the sars-cov-2 main protease reveal strategy for developing dual inhibitors against m$$^{\rm pro}$$ and cathepsin l. *Sci. Adv.* **6**, (2020). 10.1126/sciadv.abe0751 PMC7725459 33158912

[44] Jin Z (2020). Structure of M$$^{\rm pro}$$ from SARS-CoV-2 and discovery of its inhibitors. Nature.

[45] Kneller DW, Phillips G, Kovalevsky A, Coates L (2020). Room-temperature neutron and X-ray data collection of 3CL M^pro^ from SARS-CoV-2. Acta Crystallographica Sect. F.

[46] Komatsu, T. S. *et al.* Drug binding dynamics of the dimeric SARS-CoV-2 main protease, determined by molecular dynamics simulation. *Sci. Rep.* **10**, 10.1038/s41598-020-74099-5 (2020). 10.1038/s41598-020-74099-5 PMC7550358 33046764

[47] Shitrit, A. *et al.* Conserved interactions required for inhibition of the main protease of severe acute respiratory syndrome coronavirus 2 (SARS-CoV-2). *Scientific Reports* **10**, 10.1038/s41598-020-77794-5 (2020). 10.1038/s41598-020-77794-5 PMC7704658 33257760

[48] Suárez, D. & Díaz, N. Sars-cov-2 main protease: a molecular dynamics study. *JCIM* **0**, null (2020). 10.1021/acs.jcim.0c00575 32678588

[49] Gasteiger E (2003). ExPASy: the proteomics server for in-depth protein knowledge and analysis. Nucleic Acids Res..

[50] Kell, G. S. Density, thermal expansivity, and compressibility of liquid water from 0$$^\circ $$ C to 150$$^\circ $$ C. Correlations and tables for atmospheric pressure and saturation reviewed and expressed on 1968 temperature scale. *J. Chem. Eng. Data* **20**, 97–105 (1975).

[51] Schrödinger Release 2017, N. Y., LLC. LigPrep (2017).

[52] Schrödinger Suite 2017-2, N. Y., LLC. Epik, Protein Preparation Wizard (2017).

[53] Banks JL (2005). Integrated Modeling Program, Applied Chemical Theory (IMPACT). Journal of Computational Chemistry.

[54] Berman H, Henrick K, Nakamura H (2003). Announcing the worldwide Protein Data Bank. Nature Struct. Biol..

[55] Madhavi Sastry, G., Adzhigirey, M., Day, T., Annabhimoju, R. & Sherman, W. Protein and ligand preparation: parameters, protocols, and influence on virtual screening enrichments. *J. Comput.-Aided Mol. Des.* **27**, 221–234 (2013). 10.1007/s10822-013-9644-8 23579614

[56] Friesner RA (2006). Extra precision glide: docking and scoring incorporating a model of hydrophobic enclosure for protein-ligand complexes. J. Med. Chem..

[57] Halgren, T. A. *et al.* Glide: a new approach for rapid, accurate docking and scoring. 2. Enrichment factors in database screening. *J. Med. Chem.* **47**, 1750–1759 (2004). 10.1021/jm030644s 15027866

[58] Sherman W, Beard HS, Farid R (2006). Use of an induced fit receptor structure in virtual screening. Chem. Biol. Drug Des..

[59] Zhong H, Tran LM, Stang JL (2009). Induced-fit docking studies of the active and inactive states of protein tyrosine kinases. J. Mol. Graphics Model..

[60] Wang H, Aslanian R, Madison VS (2008). Induced-fit docking of mometasone furoate and further evidence for glucocorticoid receptor 17$$\alpha $$ pocket flexibility. J. Mol. Graph. Model..

[61] Luo H-J, Wang J-Z, Deng W-Q, Zou K (2013). Induced-fit docking and binding free energy calculation on furostanol saponins from Tupistra chinensis as epidermal growth factor receptor inhibitors. Med. Chem. Res..

[62] Jacobson, M. P. *et al.* A hierarchical approach to all-atom protein loop prediction. *Proteins: Struct. Funct. Bioinform.* **55**, 351–367 (2004). 10.1002/prot.10613 15048827

[63] Jacobson MP, Friesner RA, Xiang Z, Honig B (2002). On the role of the crystal environment in determining protein side-chain conformations. J. Mol. Biol..

[64] Buscemi, S., Pace, A., Palumbo Piccionello, A. & Vivona, N. Synthesis of fluorinated first generation starburst molecules containing a triethanolamine core and 1,2,4-oxadiazoles. *J. Fluorine Chem.* **127**, 1601–1605 (2006).

[65] Martorana, A., Palumbo Piccionello, A., Buscemi, S., Giorgi, G. & Pace, A. Synthesis of 4(5)-phenacyl-imidazoles from isoxazole side-chain rearrangements. *Organic Biomol. Chem.* **9**, 491–496 (2011). 10.1039/c0ob00511h 21069129

[66] Pibiri I (2010). Fluorescent Hg^2+^ sensors: synthesis and evaluation of a tren-based starburst molecule containing fluorinated 1,2,4-oxadiazoles. Eur. J. Organic Chem..

