# Supplementary material for: The dimer-monomer equilibrium of SARS-CoV-2 main protease is affected by small molecule inhibitors
Source: Sci Rep. 2021 Apr 29;11:9283. doi: 10.1038/s41598-021-88630-9 (PMC8085067; doi:10.1038/s41598-021-88630-9)
Supplement: Supplementary file 1 — Supplementary information. [file 41598_2021_88630_MOESM1_ESM.pdf]

Supplementary Information

The dimer-monomer equilibrium of SARS-CoV-2 main protease is affected by small molecule inhibitors

Lucia Silvestrini[1], Norhan Belhaj[1], Lucia Comez[3], Yuri Gerelli[2], Antonino Lauria[4], Valeria Libera[5], Paolo Mariani[2], Paola Marzullo[4], Maria Grazia Ortore[2], Antonio Palumbo Piccionello[4], Caterina Petrillo[5], Lucrezia Savini[1], Alessandro Paciaroni[5*] and Francesco Spinozzi[2*]

[1] Marche Polytechnic University, Department of Life and Environmental Sciences and New York-Marche Structural Biology Center (NY-MaSBiC), Ancona, 60131, Italy

[2] Marche Polytechnic University, Department of Life and Environmental Sciences, Ancona, 60131, Italy

[3] CNR-IOM c/o University of Perugia, Department of Physics and Geology, Perugia, 06123, Italy

[4] University of Palermo, STEBICEF Department, Palermo, 90128, Italy

[5] University of Perugia, Department of Physics and Geology, Perugia, 06123, Italy

[*] alessandro.paciaroni@unipg.it, f.spinozzi@univpm.it

$C_\circ$ (μM)	T (°C)	κC_N (μM)	x_1	B (10^{-4} cm^{-1})	r_0 (Å)	D	ξ (Å)
3.0	15	0.54±0.03	0.46±0.03	1.5±0.1	35±2	1.19±0.05	2500±900
3.0	30	0.57±0.04	0.54±0.02	1.4±0.1	37±3	1.24±0.07	2400±800
10.0	15	1.67±0.07	0.61±0.03	1.9±0.2	53±5	1.00±0.05	2400±700
10.0	25	1.7±0.1	0.68±0.04	1.7±0.1	51±7	1.00±0.05	2400±800
10.0	30	1.89±0.09	0.80±0.03	2.29±0.09	64±5	1.0±0.1	2500±900
10.0	37	1.58±0.09	0.66±0.03	0.40±0.03	44±3	1.13±0.09	2600±800
10.0	45	1.8±0.1	0.74±0.02	0.42±0.04	48±3	1.5±0.2	2400±800
20.0	15	3.6±0.2	0.77±0.02	0.41±0.04	62±9	1.00±0.05	2400±900
20.0	30	3.6±0.2	0.86±0.02	0.79±0.06	55±7	1.00±0.09	2000±1000
20.0	37	3.7±0.1	0.91±0.02	1.13±0.08	52±3	1.18±0.05	2400±900
20.0	45	3.73±0.07	0.53±0.03	0.8±0.1	53±4	1.2±0.1	2400±1000
30.0	15	5.5±0.3	0.67±0.02	1.03±0.09	68±9	1.00±0.05	2400±900
30.0	25	5.5±0.3	0.75±0.03	1.15±0.04	67±3	1.00±0.08	2500±900
30.0	30	5.335±0.008	0.84±0.03	1.3±0.1	61±3	1.04±0.07	2300±900
30.0	37	5.9±0.2	0.83±0.02	0.33±0.02	66±5	1.3±0.1	2400±900
30.0	45	5.2±0.4	0.91±0.01	0.35±0.03	54±7	1.2±0.1	2400±900

Table S1: Single-curve parameters of the analysis of SAXS data for SARS-CoV-2 M^{pro} samples without inhibitors.

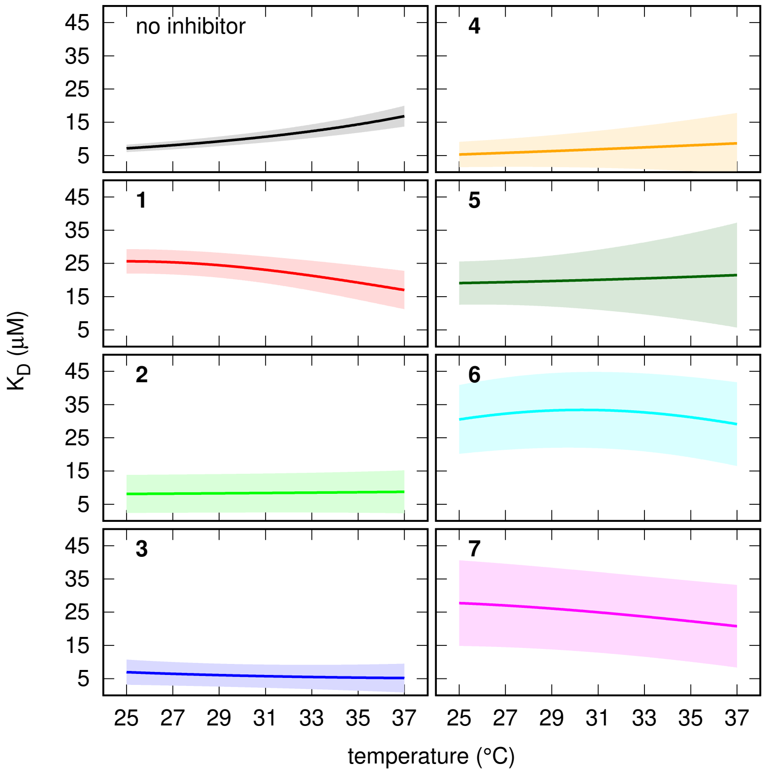

Figure S1: Temperature trends of the equilibrium dissociation constant K_D of SARS-CoV-2 M^{pro} in the absence (top left panel) and in the presence of the 7 inhibitors. Curves have been calculated on the basis of equations 2 and 4 using the thermodynamic parameters derived by the analysis of the SAXS data (Tables 1 and 3). In each panel the shaded band reports the standard deviations calculated from the propagation of the uncertainties of the thermodynamic parameters.

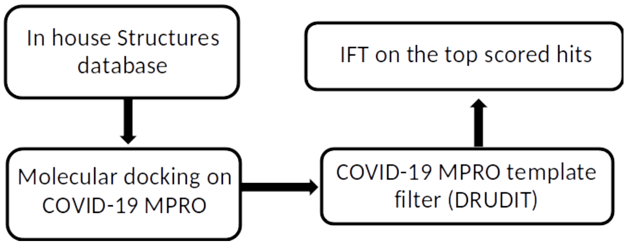

Figure S2: Flowchart of the *in-silico* inhibitor selection.

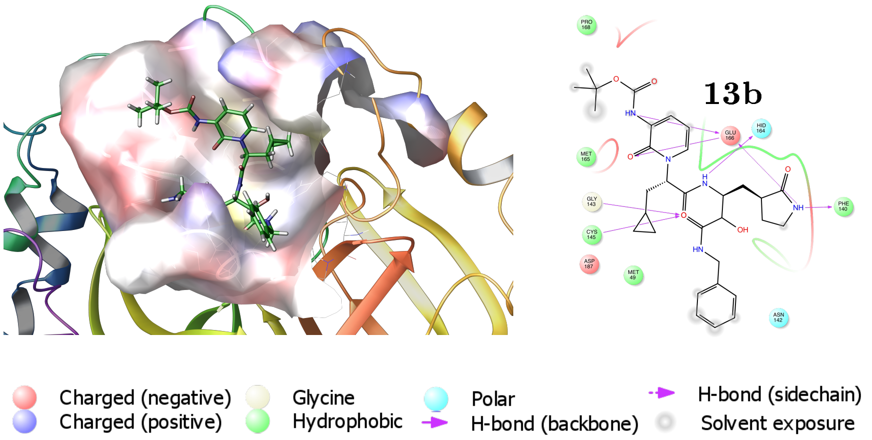

Figure S3: **Left**: 3D binding site of SARS-CoV-2 M^{pro} in complex with inhibitor **13b** (PDB code 6y2f). **Right**: amino acids map of **13b** binding active site. The picture is elaborated by Maestro Schrödinger, version 10.2 (2017)[?] .

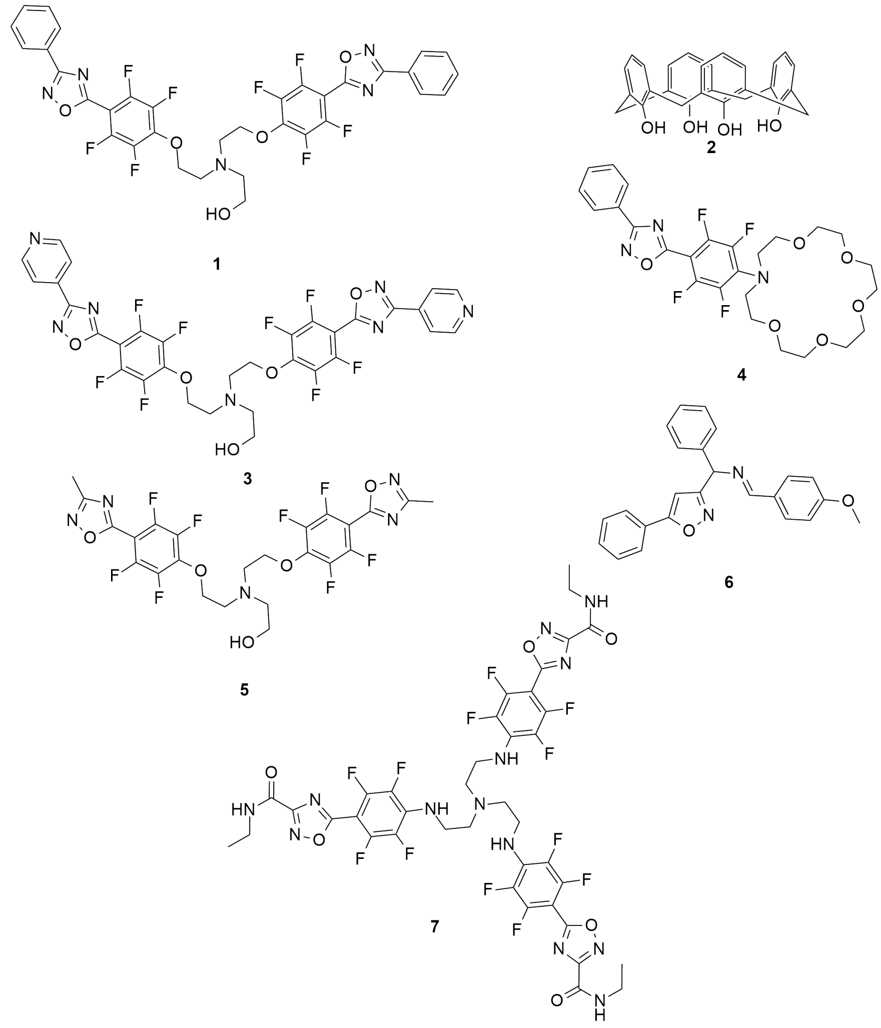

Figure S4: The best seven inhibitors molecules found by the IFD method.

Inhibitor	C_I (μM)	T (°C)	κC_N (μM)	x_1	B (10^{-4} cm^{-1})	r_0 (Å)	D	ξ (Å)
**6**	30	30	24.5±0.5	0.45±0.02	0.03±0.07	47±3	1.87±0.05	3130±20
**6**	30	37	23.7±0.6	0.49±0.03	0.0±0.1	46±1	1.91±0.05	3120±40
**6**	30	45	22.9±0.6	0.51±0.07	0.0±0.1	44±1	2.04±0.07	3100±30
**6**	60	30	22.6±0.9	0.57±0.05	0.3±0.2	45±2	1.91±0.06	3080±40
**6**	60	37	22.0±0.8	0.55±0.07	0.9±0.1	44±2	2.0±0.2	3060±50
**6**	60	45	21±1	0.5±0.1	0.8±0.2	46±2	2.0±0.2	3000±200
**7**	30	30	24±1	0.43±0.02	0.1±0.1	51±2	1.7±0.1	3040±40
**7**	30	45	22.4±0.8	0.50±0.05	0.7±0.1	46±2	1.9±0.1	3040±30
**7**	60	30	21±1	0.53±0.05	1.6±0.3	45±2	1.7±0.1	3053±8
**7**	60	37	20.5±0.8	0.50±0.02	1.5±0.2	45±2	1.74±0.06	3080±10
**7**	60	45	19.7±0.7	0.5±0.1	2.1±0.2	46±1	1.9±0.1	3100±200
**1**	30	30	25±2	0.42±0.02	0.79±0.09	49.0±0.4	1.86±0.08	3130±30
**1**	30	37	24.8±0.5	0.44±0.01	0.3±0.2	50±1	1.95±0.04	3160±50
**1**	30	45	24±1	0.45±0.04	3.2±0.3	48±4	2.11±0.09	3200±60
**1**	60	30	24.1±0.6	0.50±0.02	0.4±0.2	46.9±0.3	2.03±0.07	3260±80
**1**	60	37	23±1	0.45±0.03	0.6±0.2	47.7±0.3	2.1±0.1	3340±100
**1**	60	45	23.0±0.7	0.35±0.06	0.8±0.2	48.0±0.6	2.42±0.08	3400±100
**2**	30	37	31±2	0.36±0.03	1.3±0.3	51.5±0.1	2.91±0.02	3600±200
**2**	30	45	26.6±0.5	0.43±0.04	1.9±0.2	51±1	2.69±0.09	3600±100
**2**	60	30	26±1	0.33±0.09	0.08±0.07	49±3	2.44±0.06	3700±200
**2**	60	37	24.2±0.6	0.34±0.07	0±0	47±2	2.4±0.1	3800±200
**2**	60	45	23.9±0.9	0.35±0.06	0.09±0.05	45.3±0.9	2.56±0.06	3900±200
**3**	30	30	32±1	0.29±0.03	2.2±0.2	51.5±0.7	2.93±0.02	4100±200
**3**	30	37	30±2	0.32±0.03	2.4±0.3	55.3±0.7	2.85±0.04	4200±200
**3**	30	45	28±2	0.38±0.08	2.6±0.3	56±2	2.7±0.1	4200±200
**3**	60	30	27±1	0.28±0.06	0.1±0.1	53±3	2.2±0.2	4300±200
**3**	60	37	26±2	0.27±0.07	0.4±0.1	51±7	2.2±0.3	4400±200
**3**	60	45	24.9±0.8	0.3±0.1	0.7±0.2	46±3	2.4±0.1	4400±200
**4**	30	30	32±2	0.30±0.03	1.8±0.1	53±1	2.921±0.009	4500±200
**4**	30	37	31±1	0.35±0.03	1.3±0.2	57.9±0.8	2.81±0.06	4500±200
**4**	30	45	28±1	0.44±0.08	1.9±0.4	60±1	2.58±0.09	4500±200
**4**	60	30	25±1	0.30±0.05	0.99±0.07	55±4	2.11±0.06	4500±200
**4**	60	37	24±2	0.35±0.06	1.2±0.2	52±3	2.01±0.07	4600±200
**5**	60	30	24.7±0.7	0.46±0.05	0.02±0.08	49±3	1.9±0.1	4600±200
**5**	60	37	24.4±0.9	0.48±0.06	0.26±0.08	48.8±0.9	1.98±0.04	4600±200
**5**	60	45	24.5±0.2	0.5±0.1	2.5±0.4	48±2	2.15±0.06	4600±200

Table S2: Single-curve fitting parameters of the analysis of SAXS data for SARS-CoV-2 M^{pro} samples with inhibitors.

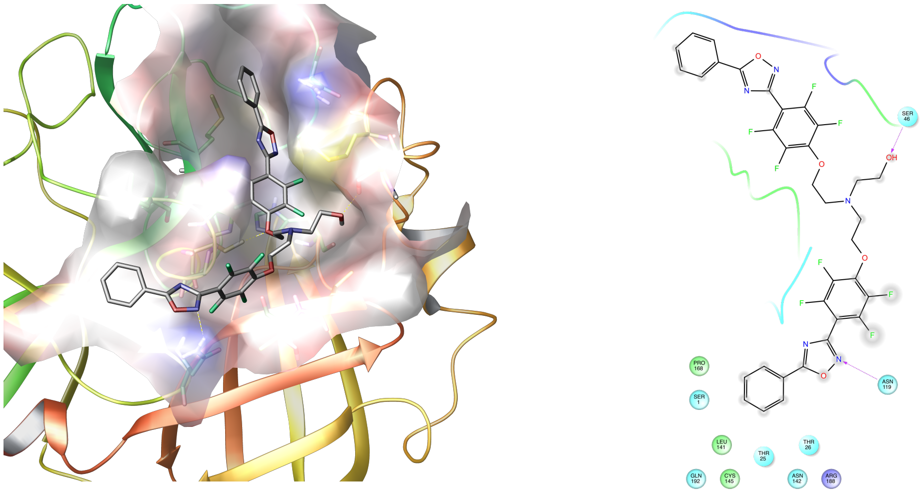

1

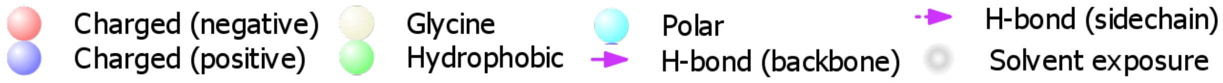

Figure S5: **Left**: 3D binding site of SARS-CoV-2 M^{pro} in complex with inhibitor **1** (PDB code 6y2f). **Right**: amino acids map of **1**. The interaction cut-off was set to 3 Å. The picture is elaborated by Maestro Schrödinger, version 10.2 (2017)[?] .

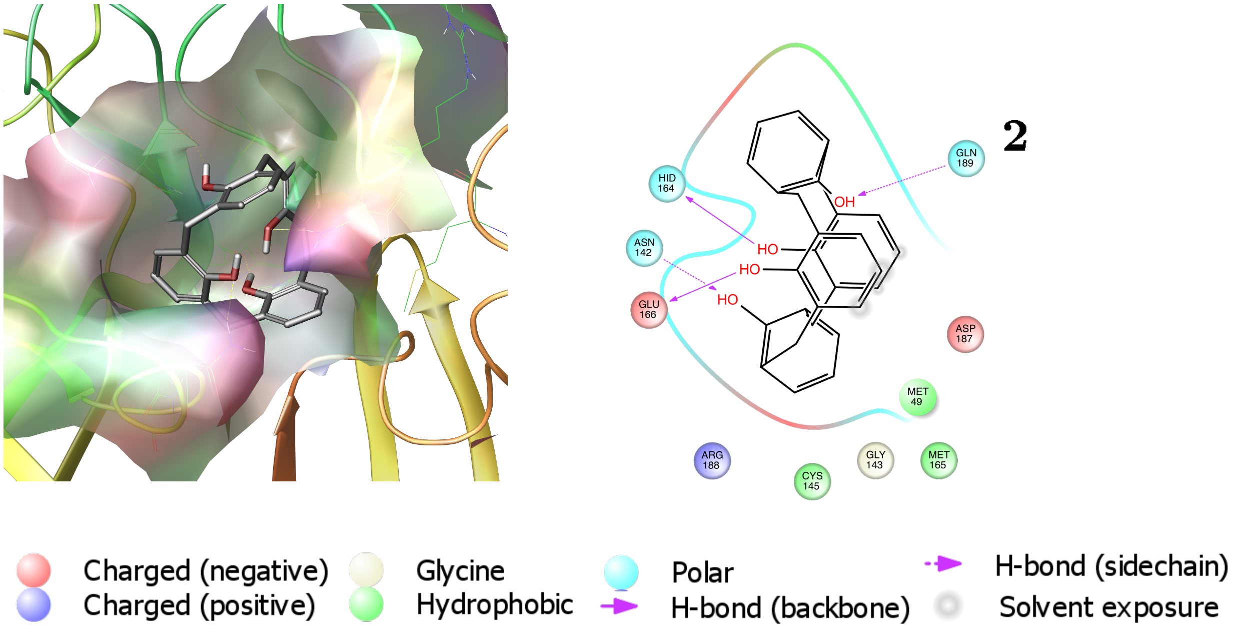

Figure S6: **Left**: 3D binding site of SARS-CoV-2 M^{pro} in complex with inhibitor **2** (PDB code 6y2f). **Right**: amino acids map of **2**. The interaction cut-off was set to 3 Å. HID represents histidine neutral δ-protonated. The picture is elaborated by Maestro Schrödinger, version 10.2 (2017)[?] .

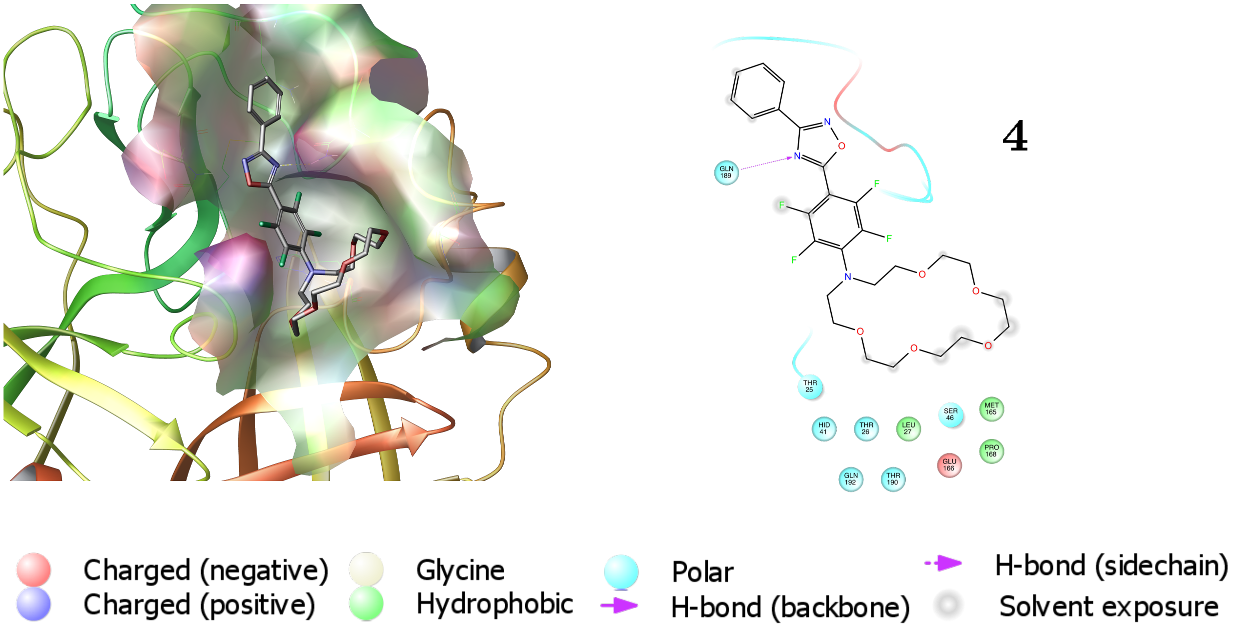

Figure S7: **Left**: 3D binding site of SARS-CoV-2 M^{pro} in complex with inhibitor **4** (PDB code 6y2f). **Right**: amino acids map of **4**. The interaction cut-off was set to 3 Å. HID represents histidine neutral δ-protonated. The picture is elaborated by Maestro Schrödinger, version 10.2 (2017)[?] .

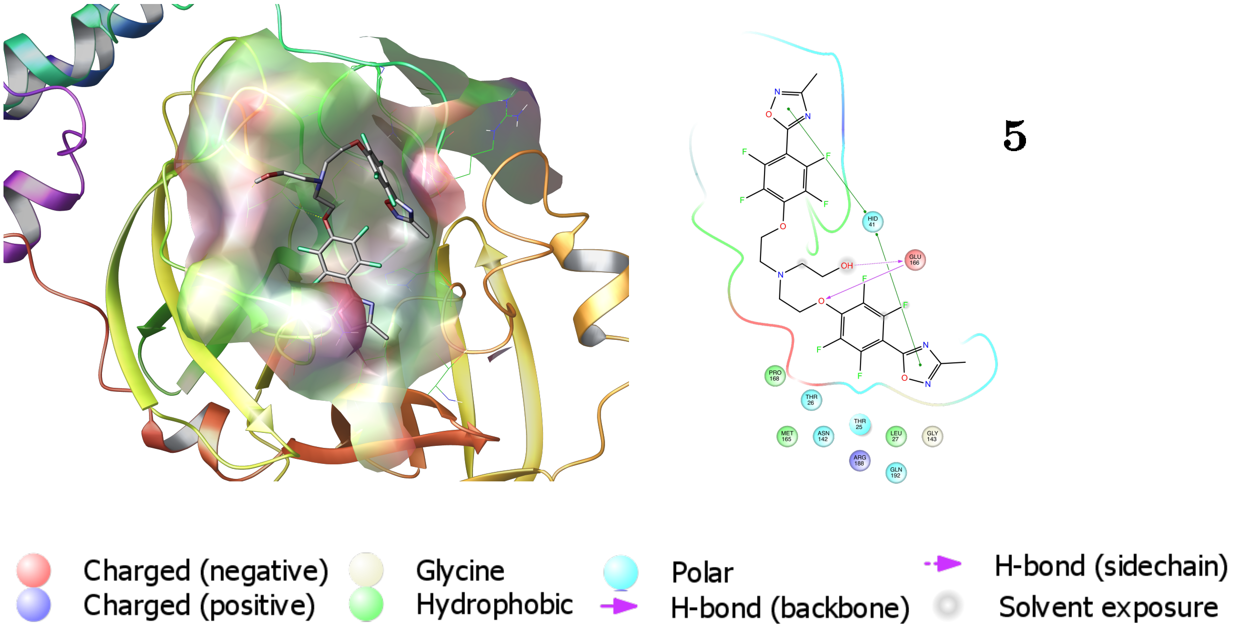

Figure S8: **Left**: 3D binding site of SARS-CoV-2 M^{pro} in complex with inhibitor **5** (PDB code 6y2f). **Right**: amino acids map of **5**. The interaction cut-off was set to 3 Å. HID represents histidine neutral δ-protonated. The picture is elaborated by Maestro Schrödinger, version 10.2 (2017)[?] .

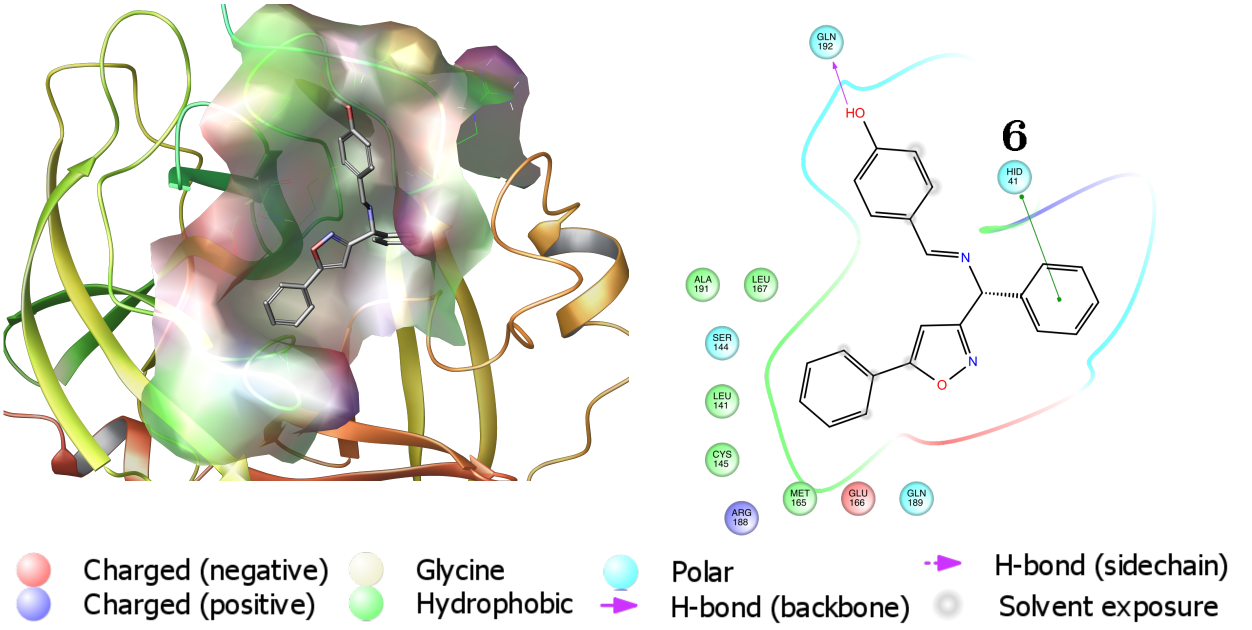

Figure S9: **Left**: 3D binding site of SARS-CoV-2 M^{pro} in complex with inhibitor **6** (PDB code 6y2f). **Right**: amino acids map of **6**. The interaction cut-off was set to 3 Å. HID represents histidine neutral δ-protonated. The picture is elaborated by Maestro Schrödinger, version 10.2 (2017)[?] .

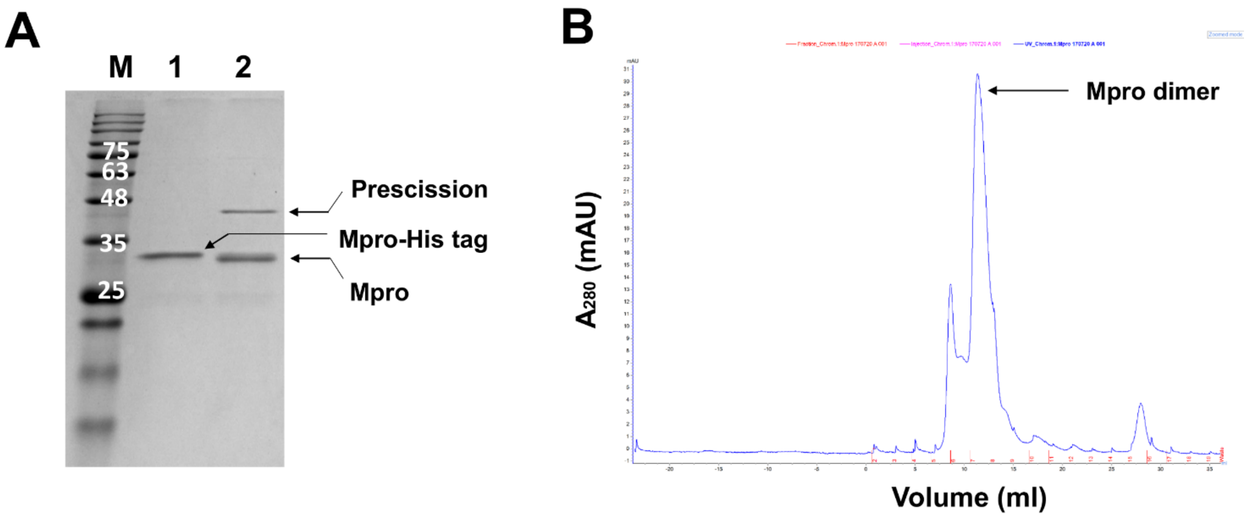

Figure S10: (**A**). SDS-PAGE showing the Prescission removal of the 6×His tag at the M^{pro} C-terminal end. (**B**). Size-exclusion chromatography profile of M^{pro} preparation at pH 7.6.

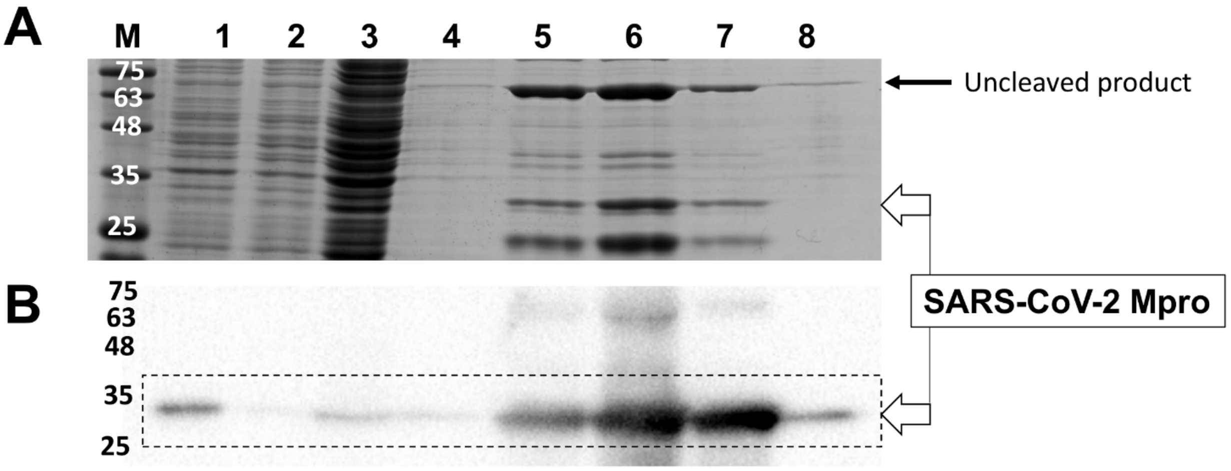

Figure S11: Small scale expression, purification and identification of SARS-CoV-2 Main Protease (M^{pro}). (**A**). SDS-PAGE of protein preparation obtained from *E. coli* BL21DE3 strains expressing M^{pro} and elution fractions from Ni-NTA affinity chromatography. M, protein ladder; Lane 1, total cell extract; Lane 2, flowthrough; Lane 3, washing 1; Lane 4, washing 10; Lane 5, elution fraction 1; Lane 6, elution fraction 2; Lane 7, elution fraction 3; Lane 8, elution fraction 4. (**B**). Western blot analysis of expressed and purified M^{pro}. M^{pro}-C-term 6×His-tag was detected by anti-His monoclonal antibody.

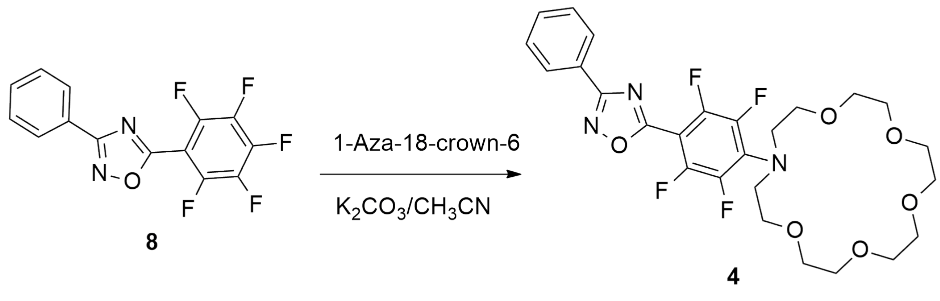

Figure S12: Synthetic procedure for the obtainment of inhibitor **4**.

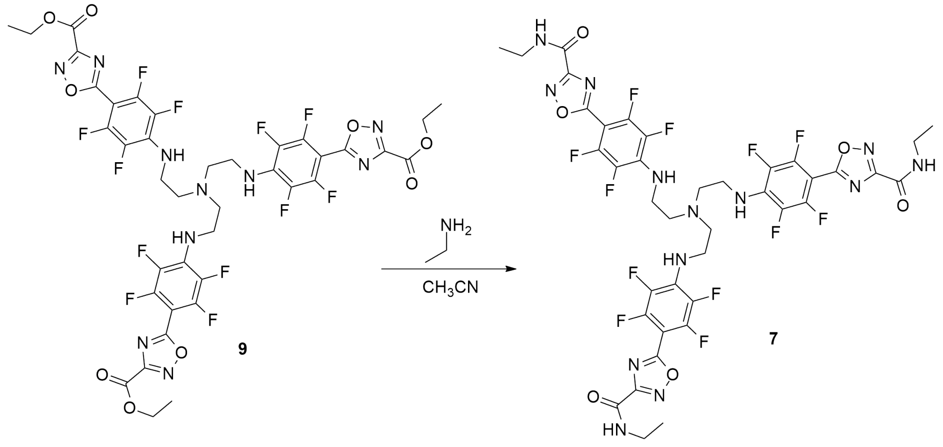

Figure S13: Synthetic procedure for the obtainment of inhibitor **7**.
